# Carbon and Phosphorus Allocation in Annual Plants: An Optimal Functioning Approach

**DOI:** 10.3389/fpls.2020.00149

**Published:** 2020-02-27

**Authors:** Marko Kvakić, George Tzagkarakis, Sylvain Pellerin, Philippe Ciais, Daniel Goll, Alain Mollier, Bruno Ringeval

**Affiliations:** ^1^ ISPA, Bordeaux Sciences Agro, INRAE, Villenave d’Ornon, France; ^2^ LSCE/IPSL, CEA-CNRS-UVSQ, Universite Paris-Saclay, Gif-sur-Yvette, France; ^3^ Signal Processing Laboratory, ICS—Foundation for Research & Technology—Hellas (FORTH), Crete, Greece

**Keywords:** allocation, optimal function, carbon, phosphorus, root-shoot

## Abstract

Phosphorus (P) is the second most important nutrient after nitrogen (N) and can greatly diminish plant productivity if P supply is not adequate. Plants respond to soil P availability by adjusting root biomass to maintain uptake and productivity due to P use. In spite of our vast knowledge on P effects on plant growth, how to functionally model enhanced root biomass allocation in low P environments is not fully explored. We develop a dynamic plant model based on the principle of optimal carbon (C) and P allocation to investigate growth and functional response to contrasting levels of soil P availability. By describing plant growth as a balance of growth and respiration processes, we optimize C and P allocation in order to maximize leaf productivity and drive plant response. We compare our model to a field trial and a set of hydroponic experiments which describe plant response at varying P availabilities. The model is able to reproduce long-term plant functional response to different P levels like change in root-shoot ratio (RSR), total biomass and organ P concentration. But it is not capable of fully describing the time evolution of organ P uptake and cycling within the plant. Most notable is the underestimation of organ P uptake during the vegetative growth stage which is due to the model's leaf productivity formalism. In spite of the model's parsimonious nature, which optimizes for and predicts whole plant response through leaf productivity alone, the optimal growth hypothesis can provide a reasonable framework for modelling plant response to environmental change that can be used in more physically driven vegetation models.

## Introduction

Plants respond to low P supply by growing more roots as they are responsible for its uptake (Marschner et al., 1996). Depressing shoot C allocation and preferential partitioning of assimilates to roots is a well documented phenomenon at low P availability (Fredeen et al., 1989; Rychter and Randall, 1994). The importance of nutrient control on C allocation between shoots and roots is even clearer under magnesium (Mg) and potassium (K) limitation, since they have a direct role in assimilate transport from leaves and can disrupt the plants' mechanism to counter reduced P availability (Cakmak et al., 1994). Once P supply is limited, plants exhibit lower leaf surface (Fredeen et al., 1989) and reduced photosynthetic capacity (Fredeen et al., 1990) all of which contribute to reduction of productivity and total plant biomass. Another important point is the stoichiometric flexibility of plant tissue as a response to environmental change (Elser et al., 2010); especially to change in nutrient availability. Since plant size and nutrient concentration are strongly connected due to the underlying machinery driving plant growth (Ågren, 2008), representing stoichiometry change is a crucial step when coupling the C and nutrient cycles.

One of the main hypotheses explaining plant response to nutrient, CO_2_ and water availability is that plants make optimal use of resources in order to maximize growth (Bloom et al., 1985; Chapin et al., 2011). Resources are acquired by the plant and distributed to its organs, all of which serve a different function (e.g., leaves for C assimilation, roots for nutrient uptake, stems for structural support). Investing into an organ will increase its capacity to perform a certain function, but will necessarily incur a cost as resources are devoted to its formation and upkeep. The plant should thus invest into an organ and maximize the organ's efficiency, which is the amount of functional gain per amount of resource invested. If we assume individual plants grow as much as they can in order to survive, they should optimize resource distribution to maximize growth. Applying this principle to the problem of nutrient limitation, a plant should grow roots (which take up nutrients) in such a way that uptake efficiency is highest (or the least amount of roots needed to satisfy the growth requirements). Consequently, at different nutrient availabilities the plant will exert more or less effort in growing roots and thus change its root-shoot balance.

The question of how to model C allocation due to nutrient limitation is a long standing one (Ågren and Wikström, 1993; Thornley, 1995; Franklin et al., 2012) and the concept of optimal resource distribution would fall in between functional balance and teleonomic ones. Functional balance states that allocation of C towards an organ will be driven by its function (C assimilation in leaves, P uptake in roots) whereas teleonomic states that allocation is guided with a specific goal in mind, which is maximizing productivity as proxy of individual fitness. The combination of these two results in an approach called optimal functioning (OF), which has shown promise in explaining plant response to a changing environment (McMurtrie et al., 2008; Mäkelä et al., 2008; Dewar et al., 2009; Franklin et al., 2009). By describing plant growth as a balance of growth and respiration processes, the concept of optimizing (or maximising) productivity provides a general framework for describing plant response to nutrient availability (Dewar et al., 2009). Furthermore, some avenues to improve this approach are proposed (Dewar et al., 2009) such as: extension to different time-scales, inclusion of multiple resources (energy, water, nutrients, etc.) and developing practical methods to be included in dynamic global vegetation models (DGVM).

Previously mentioned OF approaches (Dewar et al., 2009) deal with steady state vegetation growth. Even though this is a robust representation of ecosystem response, a dynamical representation of the underlying processes (via a DGVM) is warranted as vegetation growth is inherently seasonal. One of the main goals of DGVMs (Krinner et al., 2005) is to bridge the gap between the fast (order of 1 h) hydrologic and biophysical processes of water and energy exchange on one hand and slow (order of 1 year and more) vegetation dynamics like fire, sapling establishment, light competition, tree mortality on the other. This is achieved with the use of physical parametrizations of C exchange through photosynthesis (Collatz et al., 1992; Farquhar et al., 2001) stomatal conductance (Ball et al., 1987) and respiration models (Parton et al., 1988; Ruimy et al., 1996) which drive vegetation growth and its interaction with the biosphere on a daily time step.

The OF approaches mentioned so far (Dewar et al., 2009) have focused on nitrogen (N) only. This is understandable as N is the principal nutrient required for plant structure and metabolism functioning. Here, we focus on P because its effects on plant productivity can extend to the ecosystem level, where it has been shown (Elser et al., 2007) that P has a similar potential to N across terrestrial biomes. Furthermore, unlike N, soil P transport and plant uptake are mainly determined by diffusion due to poor mobility of P in soils (Barber, 1995; Hinsinger et al., 2011). Increasing root volume through root proliferation is one of the main ways of combating P limitation (Plaxton and Lambers, 2015) making root investment (and subsequently whole plant response) an important factor to consider. Representing the effects of P limitation and especially how it affects root growth is thus as important as N, if plant response to a changing environment is to be investigated.

Modelling of plant P limitation and its systemic effects has been topic of considerable research in natural (Wang et al., 2010; Goll et al., 2012; Yang et al., 2014; Goll et al., 2017; Yang et al., 2019) and agricultural systems (Jones et al., 1984; Daroub et al., 2003; Delve et al., 2009; Dzotsi et al., 2010). While the scope of these models differ, a common thread in all of them is still a rather empirical approach to describing plant P limitation (Franklin et al., 2012; De Kauwe et al., 2014). In these, C allocation typically relies on fixed allometric ratios or prescribed functional relationships with respect to resource availability (Franklin et al., 2009) that encounter difficulties in reproducing shifting allocation patterns (De Kauwe et al., 2014). OF could supplement these P modelling efforts, as it provides a more mechanistic foundation to C allocation that could potentially tackle these short-comings (Dewar et al., 2009; Franklin et al., 2009; De Kauwe et al., 2014).

To this end, we propose a model which optimizes plant growth according to P availability as a balance of leaf productivity, root P uptake and organ respiration on a daily time step. The goal is a general description of plant response to P availability like change in root-shoot ratio, stoichiometry and total biomass. We present an optimization tool (Dantzig and Thapa, 1997) which allows for dynamic (day-by-day) modelling and could be extended to include other growth limiting resources, as well as implemented within more physically based vegetation models (like DGVMs; Krinner et al., 2005).

## Methods

### Modelling Framework

We consider only C and P availability in eliciting plant response, with all other growth resources assumed to be non-limiting. Leaves assimilate C and roots take up P on a daily basis. Although C is the main resource for biomass growth, P is needed to sustain the plant's metabolism. Assuming that individual plants try to maximize growth, a balance between the amount of leaf and root should be established according to P availability. The problem can be translated into a optimal resource use one: given a limited supply of C and P, how can they be distributed to maximize plant growth? To solve this problem we employ linear programming (Dantzig and Thapa, 1997) a method which calculates the best possible outcome given a problem stated by a system of linear equations. Linear programming is a well established theory used in many areas of operational research (Dantzig and Thapa, 1997) where performance of a system is maximised (like profit or units produced) given a limited amount of resources (like construction material, capital, labour, or time).

**Figure 1:**
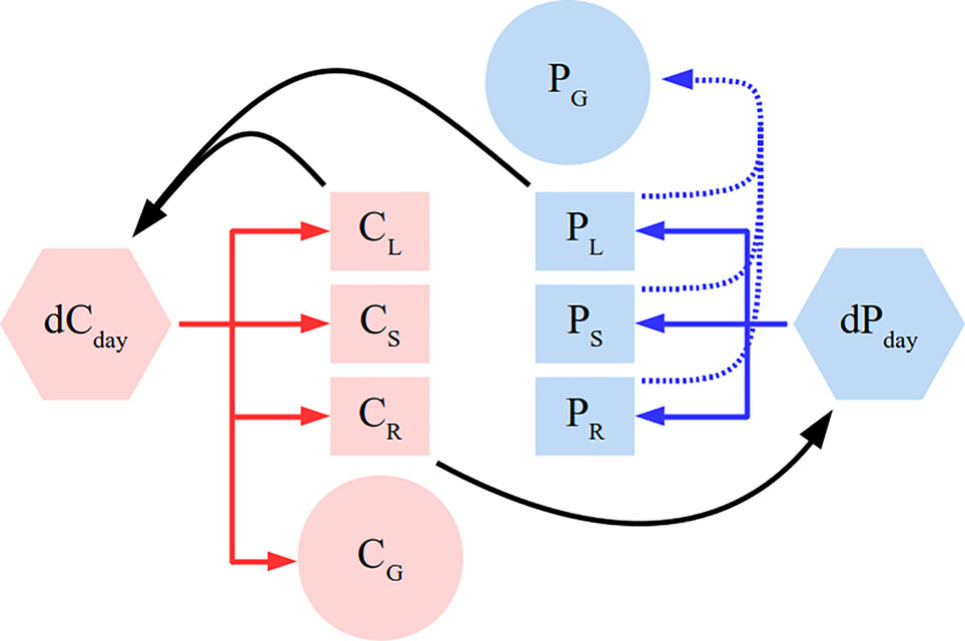
Simplified schema of the plant model. Full arrows depict C (red) and P (blue) flux directions. Black color depicts feedbacks of different pools on gross productivity (*dC_day_*) and root P uptake (*dP_day_*). Different letters correspond to leaf (L), root (R), stem (S), and grain (G). Dashed blue line represents grain P remobilisation. The model additionally contains C respiratory fluxes and allometric constraints, which were not depicted here for clarity sake and are detailed in the *Methods* section.

The plant is modeled as a system of linear mass-balance equations (model schema in **Figure 1** describing assimilation, root uptake and respiration. The plant consists of several organs: roots, leaves, stem, and grain. Leaves take up C and roots take up P on a daily basis. We allocate C and P to grow plant organs as much as possible while respecting constraints in form of allometric relationships. For root P uptake, the model does not describe external physical mechanisms that determine P supply like soil diffusion. Instead, P availability is determined by the root P uptake rate which is simply the amount of P taken up per unit of root biomass per time. Stated as a linear program, the maximisation objective at each time-step is following:

(1) $$Maximize\;dC_{day}$$

where *dC_day_* is daily gross productivity, which is the amount of new C that can be allocated to different pools. *dC_day_* is proportional to leaf biomass $\left(C_{L}^{*}\right)$ via the assimilation rate (*k_CL_*) and is weighted by the leaf's light (*LUE*) and phosphorus (*PUE*) use efficiency:

(2) $$dC_{day}=k_{CL}\cdot dt\cdot C_{L}^{*}\cdot LUE\cdot PUE$$

(3) $$LUE=\frac{C_{L,max}}{C_{L}^{*}+C_{L,max}}$$

(4) $$PUE=\frac{P_{L}^{*}}{P_{L}^{*}+C_{L}^{*}\cdot \rho_{max}}$$

*LUE* represents saturated assimilation *via* leaf shading (Thornley, 1976) whereas *PUE* represents saturated photosynthetic capacity due to leaf P concentration (Fredeen et al., 1990). *C_Lmax_* and *ρ_max_* are the leaf dry weight and P concentration at which *LU E* and *PU E* reach half of their maximum efficiency, respectively. Equations 2–4 are a standard hypothesis in OF approaches (Dewar et al., 2009) and are the main driver of plant response to P availability. $C_{L}^{*}$ and $P_{L}^{*}$ are future leaf C and P pools, respectively. Other pools which exist, but are not photosynthetically active are roots (R), stems (S), and grain (G). Future pool refers to updating the current one at every time-step. For C, updated pools are a balance (Eq. 5) between C allocation (Eq. 6) and respiration (Eq. 7):

(5) $$C_{i}^{*}=C_{i}+dC_{i}-rC_{i}$$

(6) $$dC_{day}=\sum_{i=L,R,S,G}dC_{i}$$

(7) $$rC_{i}=\lambda_{C,i}\cdot C_{i}\cdot dt$$

The variables here are $C_{i}^{*}$ (future C pool), *dC_i_* (amount of C allocated), and *rC_i_* (respired C). *C_i_* (current C pool) is a state variable and $\lambda_{C_{i}}$ (respiration rate) is time-invariant. Index *i* denotes different organs. In the case of P, future pools are a balance (Eq. 8) of allocation of P taken up by roots (Eq. 9) as well as grain P remobilisation (Eqs. 10 and 11):

(8) $$P_{i}^{*}=P_{i}+dP_{i}-mP_{i}$$

(9) $$k_{PR}\cdot C_{R}^{*}=\sum_{i=L,R,S}dP_{i}$$

(10) $$\begin{array}{cc} mP_{i}=k_{mP_{i}}\cdot P_{i}\cdot dC_{G}; & i=L,R,S \end{array}$$

(11) $$\sum_{i=L,R,S,G}mP_{i}=0$$

The variables are: $P_{i}^{*}$ (future P pool), *dP_i_* (P increment) and *mP_i_* (remobilised P). *P_i_* (current P pool) is a state variable while *k_PR_* (root P uptake rate) and $k_{mP_{i}}$ (remobilised P fraction) are time-invariant. We assume no saturating effect for root uptake (Eq. 9) to reduce model complexity, assuming it can be due to external physical limitations to P supply (eg. soil diffusion) which are not modeled here. P remobilisation flux (Eq. 10) is proportional to the amount of C going towards the grain (*dC_G_*) and the fraction of the remobilisable $\text{P}(k_{mP_{i}}\cdot P_{i})$. Allometric constraints are applied on stem and grain C filling (Eq. 12, 13) as well as on P filling of photosynthetically non-active tissue (Eq. 14):

(12) $$f_{C_{S}}\cdot dC_{L}=dC_{S}$$

(13) $$f_{C_{G}}\cdot rC_{S}=dC_{G}$$

(14) $$\begin{array}{cc} f_{P_{i}}\cdot dP_{L}=dP_{i}; & i=R,S \end{array}$$

$f_{C_{S}}$ and $f_{C_{G}}$ are the fraction of C going towards stem and grain, and are tied to the amount of C allocated to leaf (*dC_L_*) and stem respired C (*rC_S_*) respectively. $f_{P_{i}}$ is the fraction of P going towards the photosynthetically non-active tissue (root and stem) and is tied to the amount of leaf allocated P (*dP_L_*). Equation 12 follows the principle of the pipe-theory model (Shinozaki et al., 1964). For grain (Eq. 13) we rely on the concept of Iwasa and Roughgarden (1984) where grain filling is triggered once a plant reaches its maturity. In our approach, we model grain filling as a continuous process where the grain C flow reaches its peak when the vegetative part stops growing (or stem respiration is highest). Equation 14 is a necessary assumption if we want to fill the photosynthetically non-active pools with P, since the plant does not confer any benefit from doing so. Details on the various variables and parameters are given in **Table 1**.

**Table 1:** Model variables and parameters. Units correspond to values that would be expected in an agricultural field trial.

Variable	Description	Unit
*dC_day_*	Daily gross productivity	tDW ha^–1^
*dC_day_*	Daily root P uptake	kgP ha^–1^
*C_i_*	Current C pool	tDW ha^–1^
*P_i_*	Current P pool	kgP ha^–1^
$C_{i}^{*}$	Updated C pool	tDW ha^–1^
$P_{i}^{*}$	Updated P pool	kgP ha^–1^
*dC_i_*	Daily C allocation	tDW ha^–1^
*rC_i_*	Daily C respiration	tDW ha^–1^
*dP_i_*	Daily P allocation	kgP ha^–1^
*mP_i_*	Daily grain remobilised P	kgP ha^–1^
Constant		
*dt*	Time step	day
*k_CL_*	Assimilation rate	tDW tDW^–1^ leaf day^–1^
*k_PR_*	Root P uptake rate	kgP tDW^–1^ root day^–1^
*C_L,max_*	LUE half-saturation point	tDW leaf ha^–1^
*ρ_L,max_*	PUE half-saturation point	kgP tDW^–1^ leaf
*f_C,i=S,G_*	C allocation fraction	tDW tDW^–1^ leaf
λ*_C,i=L,__R,__ S,__G_*	C respiration rate	day^–1^
*f_P,i=R,S_*	P allocation fraction	kgP kgP^–1^leaf
*k_mP_*_,_ *_i =R, L, S_*	Grain P remobilisation fraction	tDW^–1^ grain day^–1^

For the hydroponic studies, the units change according to the scale of the experiment to gDW pot ^–1^ for biomass and mgP pot ^–1^ for P. i denotes organs which are leaves (L), roots (R), stem (S), and grain (G). DW stands for dry weight.

### Calibration: Observations and Protocol

We use the following datasets to calibrate our model with observations: a field trial and two hydroponic studies. The field trial contains information on maize (*Zea mays*) shoot biomass and organ P uptake during a whole growing season in 1996 (with an interval of 7–20 days) and comes from a long-term experiment in Tartas, France (Plénet et al., 2000) where maize response to different P input levels was recorded (three levels, four replicates). The hydroponic studies describe early vegetative response (within 3–4 weeks) of shoot biomass, root biomass and shoot P uptake across five different P levels at experiment end. They contain seven temperate annual pasture species (Asher and Loneragan, 1967) and five cereal/legume ones (Fageria and Baligar, 1989). The pasture species are subterranean clover (*Trifolium subterranum*), barrel medic (*Medicago tribuloides*), blue lupin (*Lupinus digitatus*), smooth flatweed (*Hypochoeris glabra*), erodium (*Erodium botrys*), capeweed (*Cryptostemma calendula*), silver grass (*Vulpia myuros*), and brome grass (*Bromus rigidus*). The cereal/legume species are alfalfa (*Medicago sativa*), red clover (*Trifolium pratense*), common bean (*Phaseolus vulgaris*), rice (*Oryza sativa*), and wheat (*Triticum aestivum*).

When calibrating, we use all of the available information to constrain the model parameters (time and P level wise) in each P availability experiment (field trial or hydroponic study). This entails 13 time points across 12 different P levels (three P levels x four blocks) in the field trial and only one time point across five different P levels in each hydroponic study. During calibration for each P availability experiment, we assume the root P uptake rate *k_PR_* to be the only parameter changing among the different P levels as it reflects P availability. All of the other parameters are kept constant across different P levels, as growing conditions are assumed to be identical with respect to other main factors determining growth (temperature, light, nutrients which are not P, etc.). In this manner the obtained values should be specific to the investigated species and the environment they were grown in, where plant response depends on P availability alone. Since we are concerned with P only, the calibrated values should correspond to growth that is equally limited by other major abiotic factors (like water, light or N) across different P levels. For the hydroponic studies we assume a plant consisting of only leaves and roots without allocation of P to roots. This is because aboveground biomass is grouped into shoots in both hydroponic studies (Asher and Loneragan, 1967; Fageria and Baligar, 1989), and no root P concentration is given in Fageria and Baligar (1989).

All of the parameters were kept constant during integration and calibrated in order to minimize the prediction error until the incremental parameter change falls below 1%. To start the calibration process we provide initial guess values based on maize, and assume these are the same when calibrating other species. To calculate the assimilation rate *k_CL_* we assume leaf specific leaf area (SLA) to be around 150 cm^2^ g DW^–1^ based on our observation dataset, and refer to Kim et al. (2006) for the net assimilation rate *A_max_* = 50 *μ* mol CO_2_ m^–2^ s^–1^. This results in *k_CL_* of approximately 1.5 gDW gDW^–1^ leaf day ^–1^ if we assume dry biomass consists of 50% C. To calculate the root P uptake rate we use data from Andre et al. (1978) where average final root dry weight is 18 gDW plant^–1^ and total P taken up 1.71 gP plant^–1^ over a period of 100 days, which results in *k_PR_* of approximately 1 mgP gDW^–1^ root day^–1^. LUE half-saturation leaf biomass *C_L,max_* was estimated from Lindquist et al. (2005) where the average Beer-Lambert extinction rate *k* was measured at 0.67 LAI^–1^. If we assume half of the light intensity is lost at same LAI $\left(LAI_{LUE=1/2}=\frac{log(2)}{k}=C_{L,max}\cdot SLA\right)$ it gives a *C_L,max_* of around 1.5 tDW leaf ha^–1^ with the previously mentioned SLA. PUE half-saturation leaf P concentration *ρ_L,max_* was estimated from Jacob and Lawlor (1991) where the majority of P limitation happens in the 1.8–7.2 mmolP m^–2^ range. This translates to 0.8–3.2 mgP gDW^–1^ with previously mentioned SLA so we assume a *ρ_L,max_* value of 1 mgP gDW^–1^. For plant respiration we rely on information on a whole plant basis (De Vries, 1972; Andre et al., 1978; De Vries et al., 1979) which aggregates contributions due to growth and maintenance respiration, since we do not distinguish them neither. It can be found that maize respiration rates fall in the 0.2–0.3 day^–1^ range in optimal growing conditions so we assume: 0.30 day^-1^ for roots, 0.10 day^-1^ for leaves, 0.03 day^-1^ for the stem, and 0.01 day^-1^ for the grain. Since P concentration and final mass are a product of growth and respiration and are not easily described by C:P ratios, we manually adjust the parameters related to allocation of C and P towards stem and root as well as grain P remobilisation, until the modeled plant somewhat resembled maize growth in our observation dataset. Afterwards we rely on the calibration procedure to pinpoint the parameter values. **Table 2** provides initial guess values and references for additional clarity. To calibrate the parameters we used Scipy's optimize package (Jones et al., 2001). To solve the linear programming problem we linearise Equations 2–4 and use the package CVXOPT (Andersen et al., 2018). We integrate the model 160 days for the field trials and 30 days for the hydroponic studies. The calibrated values are compared to our initial guess estimates in **Table 3**.

**Table 2:** Parameter initial guess values. For the hydroponic studies, the units change according to the scale of the experiment to gDW pot^–1^ for biomass and mgP pot^–1^ for P.

Variable	Value	Unit	Description	Reference
*dt*	0.1	day	Time step	–
*k_CL_*	1.5	tDW tDW^–1^ leaf day^–1^	Assimilation rate	Kim et al., 2006
*k_PR_*	1.0	kg P tDW^–1^ root day^–1^	P uptake rate	Andre et al., 1978
*C_L,max_*	1.5	tDW leaf	LUE half-saturation biomass	Lindquist et al., 2005
*ρ_L,max_*	1.0	kgP tDW^–1^ leaf	PUE half-saturation concentration	Jacob and Lawlor, 1991
*λ_CR_*	0.30	day^–1^	Root respiration rate	Andre et al. (1978)
				De Vries et al. (1979)
				De Vries (1972)
*λ_CL_*	0.10	day^–1^	Leaf respiration rate	–
*λ_C__S_*	0.03*	day^–1^	Stem respiration rate	–
*λ_CG_*	0.01*	day^–1^	Grain respiration rate	–
*f_CS_*	0.5*	tDW stem tDW^–1^ leaf	Stem C filling fraction	Manually adjusted
*f_CG_*	1.0*	tDW grain tDW^–1^ leaf	Grain C filling fraction	Manually adjusted
*f_PR_*	0.1*	kgP root kgP^–1^ leaf	Root P filling fraction	Manually adjusted
*f_PS_*	0.8*	kgP stem kgP^–1^ leaf	Stem P filling fraction	Manually adjusted
*k_mPi_*	0.05*	tDW^–1^ grain day^–1^	P remobilisation coefficient	Manually adjusted
*C_L,_*_0_	0.1	tDW leaf ha^–1^	Initial leaf biomass	Manually adjusted
*P_L,_*_0_	0.1	kgP leaf ha^–1^	Initial leaf P	Manually adjusted

The asterisk symbol (*) signifies the parameter was not used during calibration of hydroponic studies. DW stands for dry weight. References on respiration rates listed for roots are the same across all other organs.

**Table 3:** Calibrated values for both the field trial and the hydroponic studies. Values for hydroponic studies are given as an average over all species.

Parameter	Initial guess	Calibrated values ± std. error	
		Field trial	Hydroponic studies
*k_CL_*	1.5	1.71 ± 0.11	1.65 ± 0.93
*K_PR_* min	1.0	0.83 ± 0.09	0.09 ± 0.69
*K_PR_* max	1.0	1.84 ± 0.20	1.52 ± 0.83
*C_L,max_*	1.5	0.90 ± 0.09	0.64 ± 0.38
*ρ_L,max_*	1.0	1.09 ± 0.09	1.56 ± 0.59
*λ_CR_*	0.30	0.14 ± 0.03	0.41 ± 0.57
*λ_CL_*	0.10	0.16 ± 0.02	0.11 ± 0.24
*λ_CS_*	0.03	0.01 ± 0.01	–
*λ_CG_*	0.01	0.02 ± 0.01	–
*f_CS_*	0.5	0.40 ± 0.05	–
*f_CG_*	1.0	2.36 ± 0.21	–
*f_PS_*	0.8	1.56 ± 0.07	–
*f_PR_*	0.1	0.04 ± 0.09	–
*k_mPL_*	0.05	0.06 ± 0.01	–
*k_mPR_*	0.05	0.25 ± 0.12	–
*k_mPS_*	0.05	0.07 ± 0.01	–
*C_L,_*_ 0_	0.1	0.01 ± 0.03	0.09 ± 0.47
*P_L,_*_ 0_	0.1	0.03 ± 0.02	0.06 ± 0.60

All parameters were kept the same across different P levels except *k_PR_* which reflects P availability (minimum and maximum provided here). Parameter description can be found in **Tables 1** and **2**. Refer to **Supplementary Tables 1** and **2** for per-species hydroponic values and a complete set of *k_PR_* values.

As we do not describe the soil-plant mechanisms leading to P limitation, we connect the modeled root P uptake rate (*k_PR_*) to the observed soil solution P concentration (*C_p_*) after the calibration has been performed. The two are connected using a Michaelis-Menten kinetic (Eq. 15). Values and units of $a_{C_{P}}$ and $b_{C_{P}}$ are given in **Supplementary Table 3**, with the results being shown in **Supplementary Figures 2** and **3**.

(15) $$k_{PR}=a_{C_{P}}\cdot \frac{C_{P}}{b_{C_{P}}+C_{P}}$$

### Sensitivity Analysis of the Modeled Response

To provide a sense of each parameter's impact on the modeled outputs we perform the method of Sobol (1993), a global and model independent variance based sensitivity analysis (Nossent et al., 2011). Here, the output model variance (*V*) is decomposed into contributions due to each parameter input (*V_i_*) and their interactions with others (*V_ij_*) which allows the calculation of Sobol indices:

(16) $$\begin{array}{ccc} S_{i}=\frac{V_{i}}{V}; & S_{ij}=\frac{V_{ij}}{V}; & S_{Ti}=S_{i}+\sum_{i\neq j}S_{ij}+\text{...} \end{array}$$

These are ratios of partial to total variance due to the parameter's main effect *S_i_* (or first order index), its interactions *S_ij_* (or second order index) or the sum of all of them together *S_Ti_* (or total index). For a detailed derivation of these indices please refer to Sobol (1993) and Nossent et al. (2011). In our case the modeled outputs were total plant biomass, total P concentration and the RSR at simulation end. Sobol sensitivity analysis was performed with the SAlib package (Herman and Usher, 2017) in the 10%–200% range of the initial guess values (**Table 2**) using 170,000 samples.

## Results

### Modeled Plant Response

**Figure 2:**
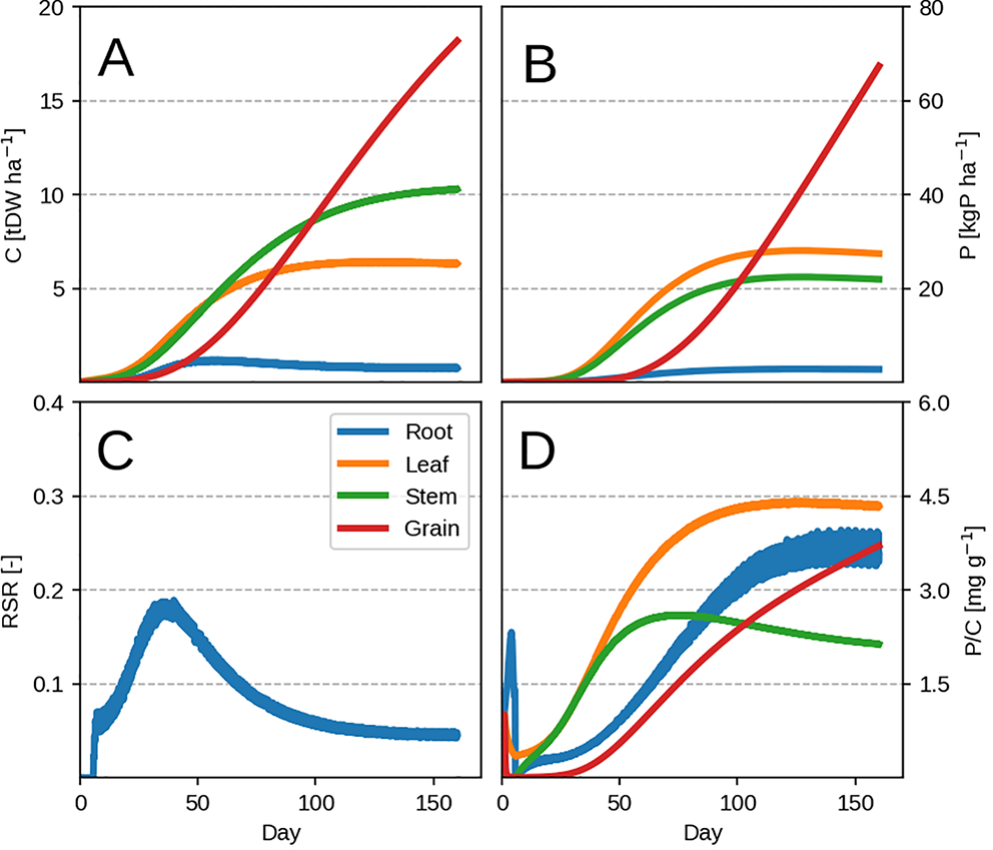
Modeled plant response with initial guess values (**Table 2**). Plots show the time evolution at high P availability (*k_PR_* = 1.0 kgP tDW^–1^ root ha^–1^ day^–1^). Upper row depicts C pools (left) and P pools (right). Panels **(A–D)** correspond to organ biomass, organ P, the plant RSR and organ P concentration respectively. Colors depict different plant organs.

The model is able to reproduce the typical growth pattern (Thornley, 1976) in annual plants: an early exponential growth transitioning into steady state at maturity, when grain filling takes place and P remobilisation occurs (**Figures 2A, B**). The evolution of organ P concentration is related to the dynamic of leaf LUE and PUE (Eqs. 3 and 4) which is reflected in the evolution of the root-shoot ratio (or RSR, **Figure 2C**). There are two distinct phases separated by the maximum growth point around 30 days. The plant initially starts to grow P poor leaves, as the biomass gain due to LUE exceeds the PUE one. As LUE decreases with self-shading, roots are progressively grown more to provide P and sustain growth. After the maximum growth point around 30 days, the plant decreases its RSR as the already accumulated leaf P can support additional growth. We provide the dynamic of changing leaf LUE and PUE in **Supplementary Figure 1** for more clarity.

Towards maturity, the RSR never falls to zero due to grain P remobilization from leaf. The leaf P remobilisation induces a decrease in PUE and is compensated by maintaining roots, which provide additional P to prevent it from decreasing further. This has the effect of increasing total P concentration due to the additional P flux into the whole plant. Roots exhibit high P concentration towards maturity (**Figure 2D**) because accumulation of P in root is driven by the amount of P going towards the leaf (Eq. 14) and not the root C balance. This means that, since the plant accumulates enough P during its lifetime to support leaf PUE, the decrease in root growth after the maximum growth point will increase its P concentration rapidly (**Figure 2D**). The “jagged” nature of C allocation (best seen in the RSR, **Figure 2C**) is due to the linear approximation of daily productivity (Eq. 2) and the optimization algorithm, which chooses to instantaneously (*dt* = 0.1 day) grow either leaf or root.

**Figure 3:**
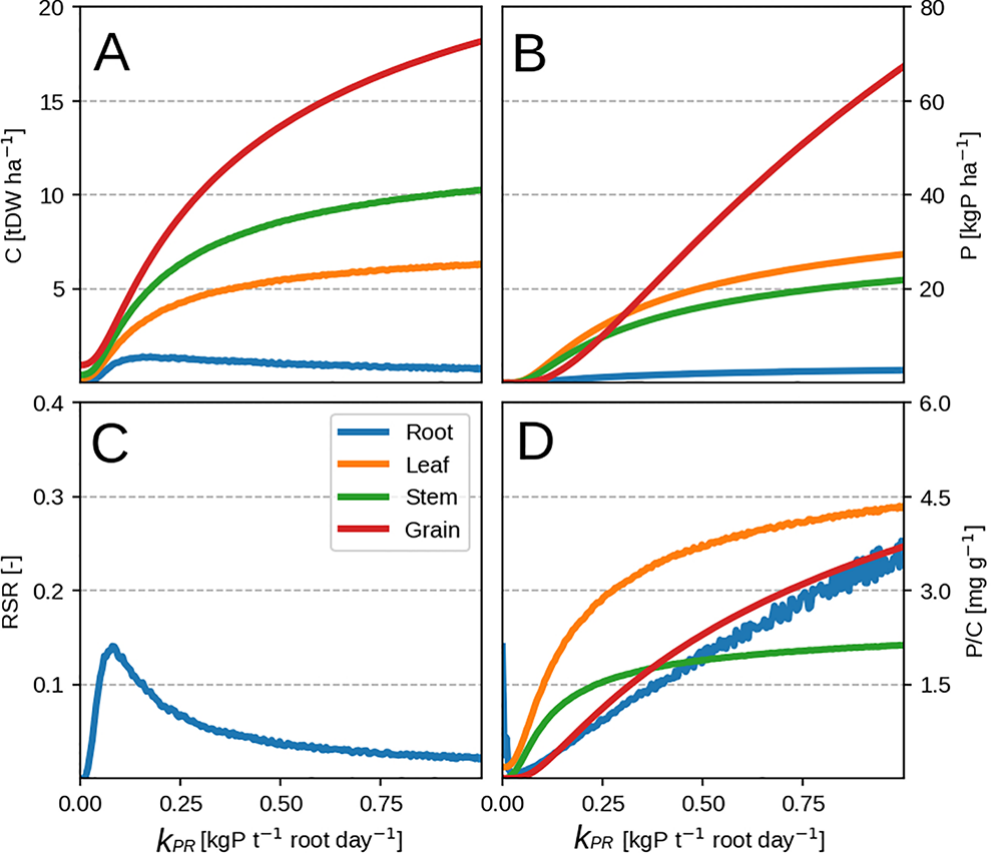
Modeled plant response with initial guess values (**Table 2**). Plots show modeled values at season end as function of root P uptake rate. Panels **(A–D)** correspond to organ biomass, organ P, the plant RSR and organ P concentration respectively. Colors depict different plant organs.

Lowering the P availability (or the root P uptake rate *k_PR_*) has the effect of decreasing total plant biomass as more C is devoted to the roots (seen by the increasing RSR in **Figure 3C**) and a decrease in leaf PUE. Across different *k_PR_* values, the RSR might seem lower than what is usually observed (0.2–0.8 in Amos and Walters, 2006). As mentioned in the first paragraph, the plant will stop investing into roots once an adequate amount of P has been accumulated (**Figure 2C**) and bring about RSR decrease. This is because (within our model) the roots' only role is active P uptake.

### Sobol Sensitivity Analysis

**Figure 4:**
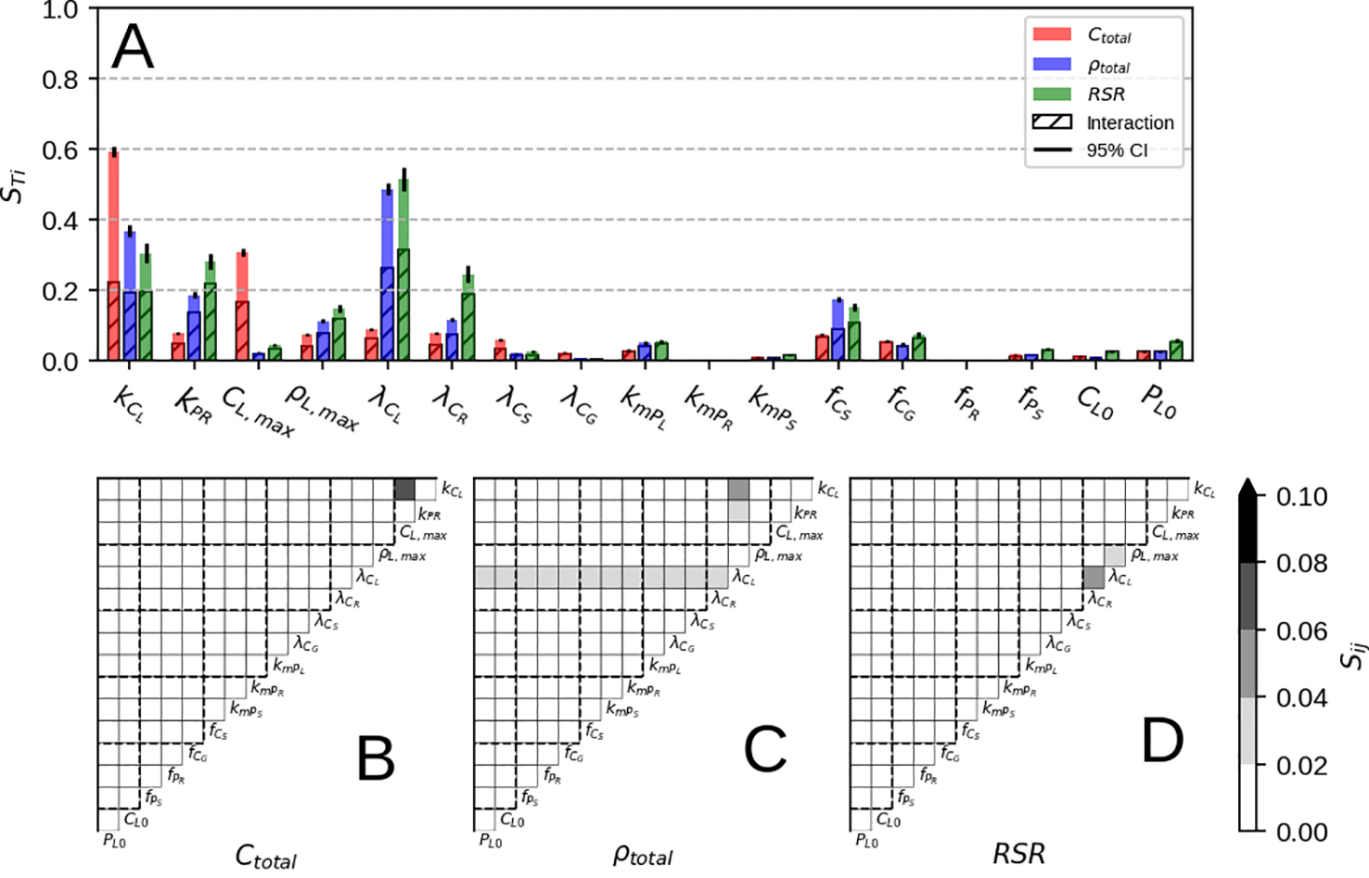
Results of Sobol sensitivity analysis on *C_total_*, *ρ_total_* and *RSR* at simulation end. Panel **(A)** shows the total contribution and the sum of all interactions for each model parameter, where as panels **(B–D)** show individual interactions. Parameter description can be found in **Table 2**.

Looking at parameter influence on final plant biomass (**Figure 4A**, red) we can see assimilation (*k_CL_*) and self-shading (*C_L,max_*) have the most direct effect, as they determine the amount of C available for growth. This fact can be also be seen in the individual interaction terms (**Figure 4B**) where *k*_*CL*_×C_*L,max*_ stands out from the rest. Other contributions come mostly from interactions by organ respiration rates (λ*_C__i_*), C allocation fractions (*f_Ci_*), root P uptake rate (*k_PR_*), and leaf P demand (*ρ_L,max_*). For λ_ci_ and *f_Ci_*, allocation of C to various organs determines the amount of lost C due to respiration; a fixed ratio for stem and grain, and a variable one for root (depending on *k_PR_* and *ρ_L,max_*)

Looking at parameter influence on final P concentration (**Figure 4A**, blue) assimilation (*k_CL_*) and leaf respiration (λ*_CL_*) exert the most direct effect. Increasing *k_CL_* increases total P concentration as more C is available for root growth, which in turn increases the flux of P towards leaf and the whole plant. Increasing λ*_CL_* brings down total P concentration on the other hand, since a higher respiration rate both raises leaf P concentration and lowers the amount of C available for root growth, in turn lowering the P flux to the leaf and ultimately the whole plant. The other parameters responsible for final P concentration are root P uptake (*k_PR_*), leaf P demand (*ρ_L,max_*), C allocation fractions (*f_C__i_*), root respiration rate (λ*_CR_*), and leaf remobilisation rate (*k_mPL_*). For *k_PR_* and *ρ_L,max_* this is not surprising, as increasing P availability and leaf P demand increases the overall flux of P into the whole plant. For *f_Ci_*, C allocation to non-root organs lowers the P flux into the plant as well P concentration due to enhanced growth. For λ*_CR_*, it determines the amount of P gained per C lost to root respiration. For *k_mPL_*, it is due to leaf PUE upkeep as explained in the previous sub-section.

Parameter influence on final RSR (**Figure 4A**, green) is similar to one obtained for P concentration (**Figure 4A**, blue) with more contribution due to parameter interaction. Also, a much higher contribution of root respiration rate (λ*_CR_*) can be seen, as it determines the net root growth rate and subsequently the plant RSR.

Parameters which seemingly do not affect neither of the three modeled quantities (**Figure 4A**) are the amount of P allocated to roots (*f_PR_*), grain respiration rate (λ*_CG_*), P remobilisation rates (*k_mPi_*) and initial leaf conditions (*C_L,0_* and *P_L,0_*). For *f_PR_*, it is due to roots' low biomass and low P allocation priority. For λ*_CG_*, it is due to the grain respiration value affecting the whole plant net C balance only slightly. For *k_mPR_* and *k_mPS_*, it is due to non-leaf remobilisation only redistributing P among different organs. *C_L,0_* and *P_L,0_* only affect the initial adjustment stage (before the max. growth point) after which biomass and P pools are a product of C and P availability.

### Comparison With Field Data

**Figure 5:**
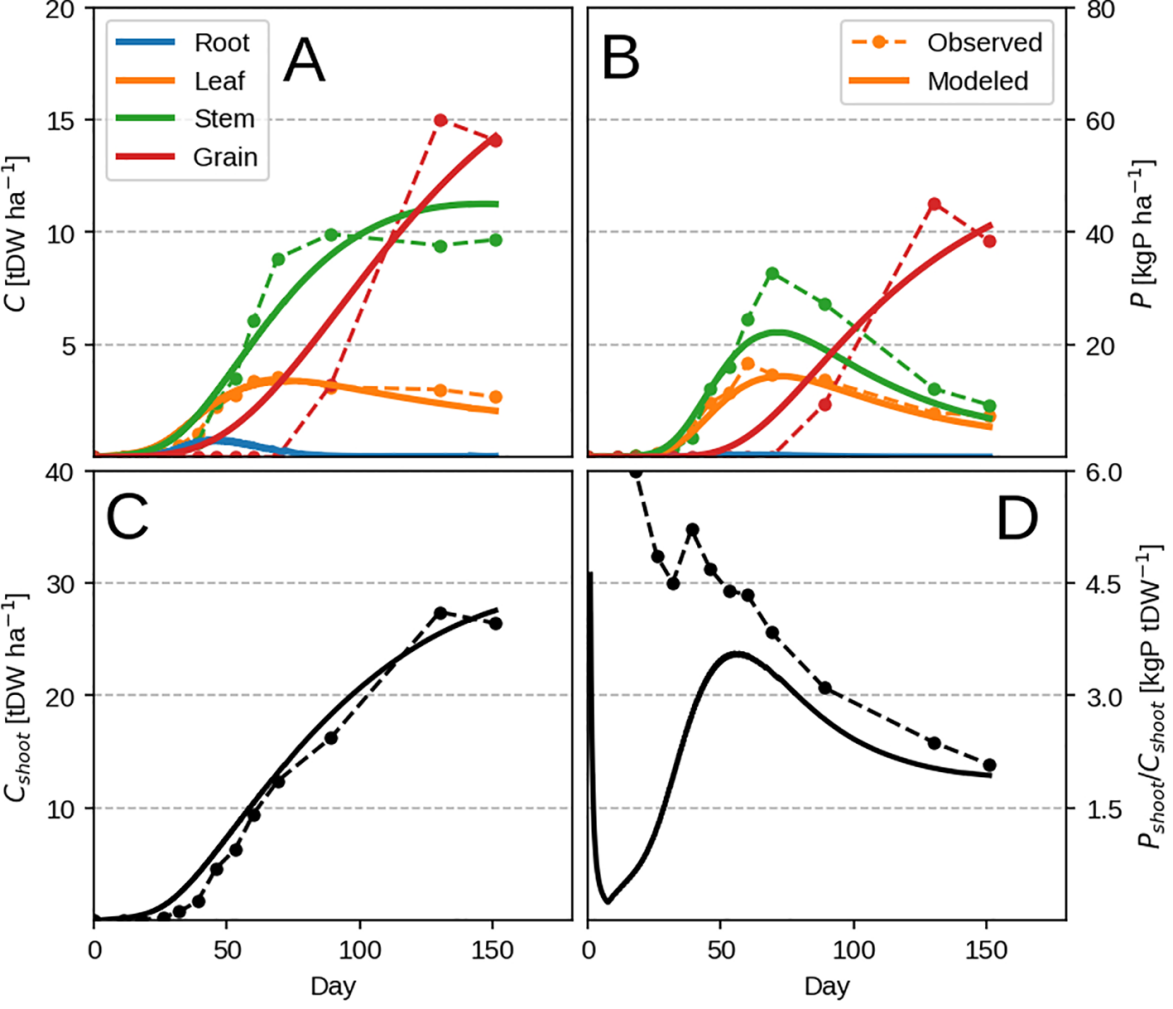
Modeled vs. observed time-series for the highest P input field maize experiment (block 4). Panels **(A, B)** show organ biomass and organ P, where as **C, D** show the same but summed across all shoot organs. Individual organs are depicted with different colors, where as total plant quantities are depicted with black.

The model is able to reproduce very well the evolution of C pools during a growing season (**Figures 5A, C**) with discrepancies probably stemming from seasonal temperature effects which are not present in our model (via respiration and photosynthesis). When it comes to the observed P pools, the model is not able to reproduce the evolution of tissue P accumulation during the vegetative stage (**Figures 5B, D**) due to the previously mentioned LUE × PUE dynamic. In the observations, it seems most of the plant P is taken up during this period and remobilised by the grain at a later stage.

**Figure 6:**
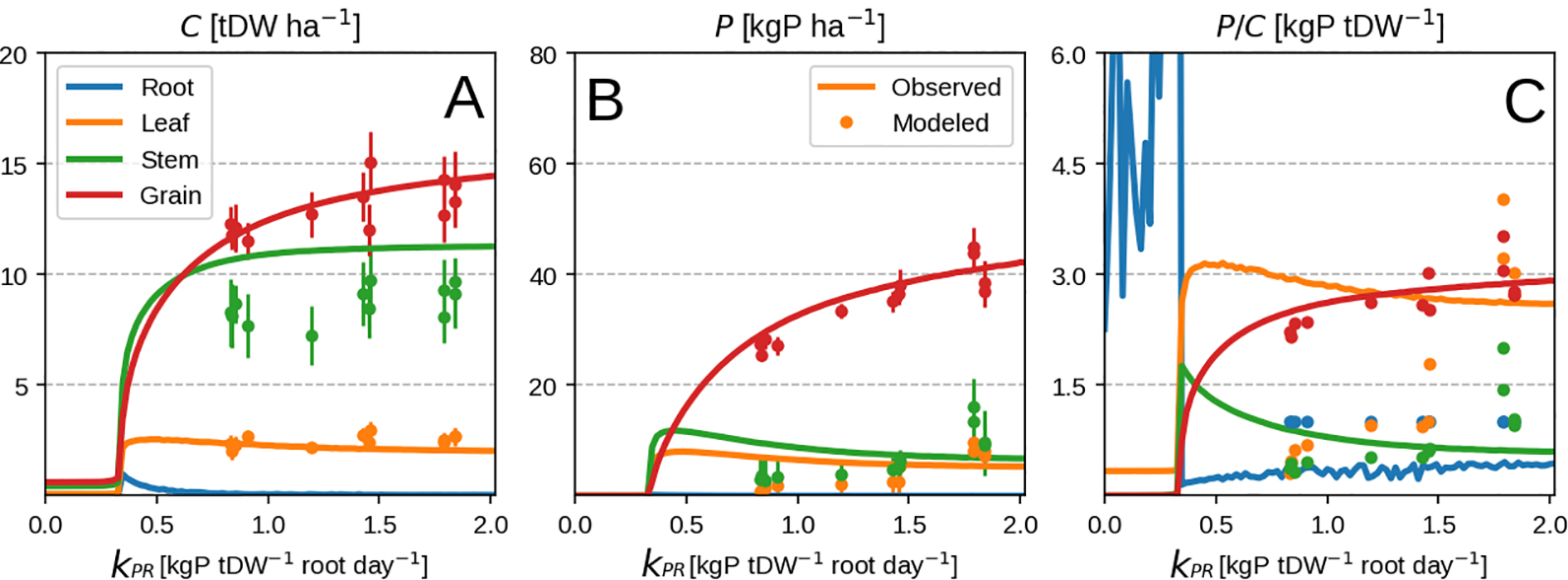
Modeled vs. observed values at field maize experiment season end as function of root P uptake rate. Leaf P remobilisation is turned on in the top row and turned off in the bottom one. From left to right the columns show C pool, P pool and concentration for leaf, stem and grain. Panels **(A-C)** correspond to organ biomass, organ P and organ P concentration respectively. Different organs are depicted with different colors. Lines are modeled values and markers are observed ones. Panels **(A–C)** correspond to organ biomass, organ P and organ P concentration respectively.

In spite of the model's inability to predict the timing of vegetative P uptake, it manages to predict well the final plant response at different P levels (**Figure 6B**). The consistency of the response can be also be seen when comparing predicted root uptake rates and measured soil P availability (**Supplementary Figure 2**). A part of the mismatch comes from the fact that we use the whole season to calibrate our model. The other mismatch comes at high P availability when luxury uptake is observed (**Figure 6C**) but is not reproducible by our method as the plant grows in the most frugal way possible. Additionally, P concentration of vegetative organs starts to decrease with higher P availability (**Figure 6C**) due to excessive grain P remobilisation. This might be due to observations falling mostly in the luxury uptake range, and could potentially be better constrained by having more P limited data.

The differences in calibrated and initial guess values (**Table 3**) are mainly due to differences in the cultivar and the growth environment. The assimilation rate (*k_CL_*) should depend on cultivar as well as temperature and the amount of light, where as LUE half-saturation point (*C_L,max_*) depends on planting density. Even though rates of root P uptake (*k_PR_*) and respiration (*k_PR_*) are set during calibration, they might not contain reliable information since they are not constrained directly. λ*_CR_* deviates much more than *k_PR_* from the initial guess during calibration because its influence on the model is lower (**Figure 4A**). But these items should not pose a serious problem as overall influence of *k_PR_* and λ*_CR_* on the model is moderate (**Figure 4A**) and the simulated root mass is not grossly misrepresented (**Figures 5** and **6**). Leaf respiration (λ*_CL_*) is much higher than the initial guess, most probably due to senescence or additional temperature dependent mechanisms which we do not account for. The same goes for stem and grain respiration rates (λ*_CS_* and λ*_CG_*) although λ*_CS_* turns out to be lower in the end. Initial leaf biomass and P values (*C_L_*_0_ and *P_L_*_0_) show a high degree of uncertainty as they do not impact the overall model behaviour, but rather the initial adjustment period until the plant reaches the maximum growth point.

### Comparison With Hydroponic Studies

**Figure 7:**
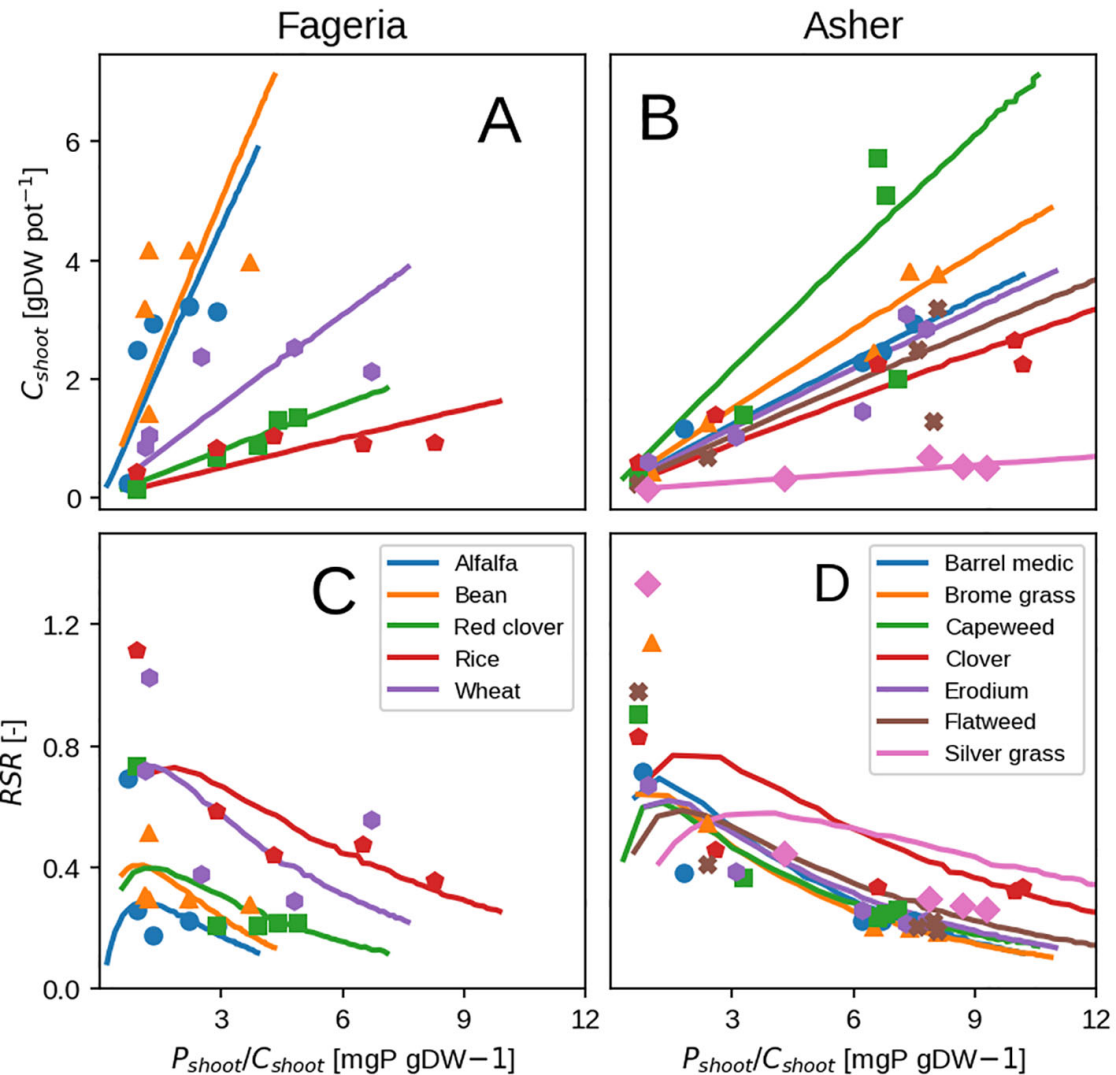
Modeled vs. observed shoot biomass (top row) and root-shoot ratio (bottom row) versus shoot P concentration. Results show the response at at hydroponic experiment end. Panels **(A, B)** show shoot biomass, where as **(C, D)** show the plant RSR Left column depicts grass species (Asher and Loneragan, 1967). Right column depicts cereals and legumes (Fageria and Baligar, 1989).

The general form of plant response to P availability is reproduced well: an increase in shoot biomass, shoot fraction and P concentration (**Figure 7**) with increasing P in the nutrient solution (**Supplementary Figure 3**). Some discrepancies remain due to toxicity effects and luxury uptake, which are present in observations but are not reproducible by the model. They can be seen at high shoot P concentration (**Figures 7A, B**) when shoot biomass remains the same or starts to decline, contrary to the model which in principle has no limit to growth. P toxicity effects are usually linked to interactions with zinc (Zn; Loneragan et al., 1979) where Zn transport from root to shoot is inhibited and Zn deficiency is induced which could be be represented in our model by a form of P control on Zn uptake. Luxury uptake of P can not be reproduced as mentioned previously, because the plant is grown in the most P efficient way possible.

The calibrated values are different from the initial guess values as expected (**Table 3**) due to species diversity and differences in the growing environment as mentioned before. The values obtained are quite similar to ones from the maize field trial (**Table 3**) with the exception of root respiration rate (λ*_CR_*) which is two to three times higher (0.3–0.6 day^–1^ for hydroponics vs. 0.14 day^–1^ for field maize). This might be due to the experiment nature that looks at initial vegetative growth only, where younger roots expend much more C. Additionally, all of the calibrated parameters show a high degree of uncertainty which is due to a relatively small number of observations (information at experiment end only).

## Discussion

According to the model results, the effects of P limitation on plants can largely be described as a compromise between root and leaf growth combined with changing efficiency of leaf P use. In situations of low P supply, plants devote relatively more C to roots at the expense of shoot to sustain plant P demand. This sets the stage for P limitation as lower leaf biomass translates into a lower biomass potential. Increasing the P availability will increase the plant's ability to grow due to higher leaf P concentration, but the efficiency of this gain decreases as more roots are needed to sustain additional growth.

The model itself does not attempt to include all of the mechanisms that modulate C and P availability which could render it into a fully-fledged vegetation one (Krinner et al., 2005; Mäkelä et al., 2008; McMurtrie et al., 2008; Franklin et al., 2009). Instead, it tries to describe plant response by relying on the functional role of different plant organs (which are acquisition of C in leaves and P in roots). These are simply expressed as resource uptake rates (the leaf assimilation rate *k_CL_* and root P uptake rate *k_PR_*) to facilitate comprehension, whereas in reality they are strongly modified by the plant's environment. Leaf assimilation depends on temperature, incoming radiation and CO_2_ partial pressure (Berry and Bjorkman, 1980) as well as water availability (Hsiao, 1973). Root P uptake depends on the properties and the amount of P in the underlying soil, as well as the physiological and physico-chemical limits to P transport (Barber, 1995). Inclusion of these processes *via* known mechanistic models like Farquhar's photosynthesis (Farquhar et al., 2001) or Barber's P diffusion one (Barber, 1995) would enable this approach to be extended to a multitude of growing environments. But the generalization and the additional level of detail would not necessarily improve the model's ability to reproduce plant behaviour which, in essence, only tries to describe how to allocate C and P between root and shoot.

Additionally, most of the model response rests on the shoulders of leaves (**Figure 4A**) which is not surprising as the whole concept of OF revolves around maximizing leaf productivity. This is a serious drawback of the approach because plants posses a multitude of architectural and development strategies to cope with reduced P availability (Niu et al., 2013; Plaxton and Lambers, 2015). Architectural ones (Niu et al., 2013) include increased top-soil foraging, lateral root growth promotion, root length density increase and cluster root formation. The first two could be modeled using a two-dimensional root model (Heinen et al., 2003) but require a vertical description of soil P fractions instead of a bulk soil P quantity. The latter two (dealing with root length density) could be attempted by implementing different root orders with varying C respiration and loss rates. Development strategies (Plaxton and Lambers, 2015) include root exudation of acids and enzymes, mycorrhizal association, lowering the metabolic cost of photosynthesis and increasing P remobilisation efficiency/seed P content among others. But these require a much more complex description of soil P chemistry, as well as a full accounting of C costs and benefits for each of the underlying processes. Another very important point is that, within this model, roots are tasked with P uptake only. The consequence of this assumption can be seen through RSR underestimation in the modeled result (**Figures 2** and **3**). In reality, roots posses much more functional role like providing structural support and maintaining transpiration (Ryan et al., 2016) which are left out as we focus on P exclusively. Nevertheless, we foresee no fundamental issues with the implementation of all of these processes, which we avoid here to facilitate comprehension of the allocation model and its results.

In spite of our model not having a very detailed root uptake module, the results presented here should not be deemed invalid due to certain processes missing (eg. root age effects or competition). At least, constant root nutrient uptake rate seems to be a sufficient requirement in explaining whole-plant response; from reproductive timing (Iwasa and Roughgarden, 1984) and root-shoot partitioning (Ågren and Ingestad, 1987; Agren, 2003) to water economy of trees (McMurtrie et al., 2008). The root module complexity could be slightly increased with a saturating function of root mass *C_R_* to mimic the two mentioned effects, and similar to what was done in Franklin et al. (2009) and Mäkelä et al. (2008). But even this should not be necessary to elicit the modeled response, as additional C gains are more and more expensive due to increasing requirement for root growth, *via* PUE saturation (Eq. 4) and respiring roots (Eq. 7). Rather than singling out any particular root related mechanism (mentioned in the previous paragraph) as an absolute necessity in modelling nutrient uptake, a more exhaustive approach (and a logical future step) would be to include multiple well known ones (Niu et al., 2013; Plaxton and Lambers, 2015) and compare their contribution relative to others; ideally using different methods of modelling C & P allocation (Ågren and Wikström, 1993; Thornley, 1995; Franklin et al., 2012).

Our approach is similar to previous OF ones (McMurtrie et al., 2008; Mäkelä et al., 2008; Franklin et al., 2009) where plant productivity is modified by leaf nutrient concentration and allocation among different organs optimized in order to reach maximum growth. This assumption provides a reasonable starting point for describing plant physiological and functional response, but overlooks the fact that leaves are the only ones impacted by the PUE assumption (Eq. 4). According to this hypothesis (which is driven by leaf concentration alone) there is no benefit from investing P into non-assimilating organs. To overcome this, allocation of P is linked *via* stoichiometry constraints (Eq. 3 in Franklin et al., 2009) or directly to the leaf concentration (Eq. 8 in Mäkelä et al., 2008);. We follow the second approach, where filling of non-assimilating organs is driven by the leaf P flux (Eq. 14). An answer to this issue might be to increase model complexity like in Thornley's transport-resistance approach (Thornley, 1995) where a plant is described as a network of nutrient and C exchanging organs, whose individual growth is determined by the labile (or exchanged) substrate concentration. But the problem using this approach is the reliance on parameters which are seldom measured (Agren, 2003) like rates of nutrient productivity, substrate utilization rates and transport resistances.

One notable difference between our model and some of the mentioned studies (Mäkelä et al., 2008; Franklin et al., 2009) is the lack of maintenance respiration due to P. Maintenance respiration is defined as a C cost deducted from gross primary productivity (GPP) to support the nutrient's metabolic activity. For N, this cost is related to protein turnover which requires energy for replacement and repair (Ryan et al., 1996) where as for P the cost could be related to formation of nucleic acid and triose phosphate (Rowland et al., 2017). We chose not to implement this process primarily because the available information is confined to forest ecosystems in tropical and sub-tropical area (Meir et al., 2001; Rowland et al., 2017) which are not studied here. On a conceptual level, this assumption is not necessary to elicit plant response to varying nutrient availability, due to the previously mentioned combination of saturating PUE and respiring roots. Introducing additional C costs due to P maintenance respiration should modify only the final C balance at different P levels, but separating P contributions to maintenance respiration is difficult at this point due to dearth of data.

In spite of some short-comings, OF approaches provide a sensible description of plant development that relies less on empirical constraints (like potential leaf-area curves, thermal sum driven phenology and prescribed allocation patterns) which are often employed in eco-physiological models (Zaehle and Dalmonech, 2011; De Kauwe et al., 2014; Rosenzweig et al., 2014). Even though seasonal dynamics of P allocation are not captured well, it might not be a critical issue as final distribution of P among plant organs should have an effect on long-term P cycling (Vitousek et al., 2010) and can be captured well by final P concentration. What is more important is the representation of C phenology, as it has a more direct connection to the plant's energy and water balance. This paves way for connection to the plant's metabolism that is strongly moderated by temperature and water availability (Hsiao, 1973; Berry and Bjorkman, 1980; Atkin, 2003).

For temperature, this should be pretty straightforward by moderating rates of assimilation (*k_CL_*, **Table 1**) and respiration (λ*_Ci_*, **Table 1**) *via* known mechanistic or empirical relationships (Berry and Bjorkman, 1980; Atkin, 2003). For water, an increased level of complexity is warranted. The main mechanism of plant response to water stress is the regulation of stomata closure to maintain internal water potential (Hsiao, 1973). Even though a multitude of physiological symptoms emerge in water stressed conditions (Hsiao, 1973) the most pertinent one is the loss in photosynthetic capacity due to a decrease in leaf CO_2_ flux with stomata closure. This presents two avenues to modify plant productivity, either by increasing root water uptake or decreasing leaf transpiration. Increasing root uptake will increase C costs due to root growth and maintenance, where as decreasing leaf transpiration will decrease C gains through lower assimilation capacity as mentioned before. One way to approach this problem is to prioritize water by prescribing stomate behavior at different water levels, and then optimize C allocation to maximize nutrient driven productivity like in (McMurtrie et al., 2008). But taking into account both of these strategies requires additional links *via* the assimilation rate *k_CL_* and the plant's water balance that can not be captured by a simple resource use kinetic typically utilised in OF approaches (Dewar et al., 2009).

So far we tacitly assume P limitation can be treated in the same way as N. Even though this is not justified physiologically, the observed functional response of root biomass change (Poorter et al., 2012) can serve as an argument for it. From a leaf productivity perspective, the primary role of N is to provide the enzymes that catalyze photosynthesis where as for P, it is the ribosomes that allow their formation (Ågren, 2008). Decreasing availability of both N and P should have an effect on leaf photosynthetic efficiency and drive plant response. This is what our PUE kinetic (Eq. 4) tries to achieve similar to the other N based studies (Dewar et al., 2009). Furthermore, their interaction could be attempted by weighing gross productivity (Eq. 2) with individual resource use kinetics ones like our PUE one (Eq. 4). It is very likely though that caveats encountered here would be even more pronounced with multiple limiting factors, since plant response is driven by leaf alone.

One of the most contentious issues in OF is its main hypothesis of productivity maximization as a proxy of fitness (Dewar et al., 2009). Plants are observed to prioritize either resource use or conservation depending on resource availability in their growing environment (Sterck et al., 2006). In productive environments, plants tend to invest mostly into resource acquisition and growth to make use of available resources. In nonproductive ones, they tend to have slow leaf and root turnover rates, as well as longer residence time of nutrients in organs to increase survival rates through resource conservation (Sterck et al., 2006). From a theoretical standpoint, evolutionary game theory has also shown the apparent infeasibility of the productivity maximization hypothesis, because it does not consider competition with neighbors (Schieving and Poorter, 1999; Dewar et al., 2009; Anten and During, 2011). In multispecies stands, these have shown that evolutionarily stable strategies (or ones that can not be outcompeted by an invading species) involve less than optimal productivity (Schieving and Poorter, 1999; Anten and During, 2011). Although, this might not be important in monospecies crop stands (which we largely focus on here) where the evolutionary pressure is to maximize grain yield, for which maximizing productivity should be the most appropriate strategy (Gutschick, 1988; Schieving and Poorter, 1999). But instead of focusing on OF as a model that reproduces viable evolutionary strategies, its strength lies in the fact that plant response is an emergent phenomenon based on relatively simple assumptions, rather than a product of a complex set of scheduling rules (McMurtrie et al., 2008; Dewar et al., 2009).

Our model could be integrated within a dynamic vegetation model (Krinner et al., 2005) where growth can be driven by a more physical description of underlying plant processes. In our approach, assimilation and root uptake are directly connected to leaf and root biomass. These could be transformed to leaf area index and root length density, making them amenable to explicit photosynthesis (Farquhar et al., 2001) and soil-root diffusion (Barber, 1995) parametrizations. Furthermore, connection to the underlying soil can be done *via* a soil P model (Wang et al., 2007; Ringeval et al., 2017). Such coupling could help us investigate effects of P limitation in ecosystems (Peñuelas et al., 2013) while accounting for plant adjustment and investigate long term effects of P cycling (Goll et al., 2012; Ringeval et al., 2017).

## Data Availability Statement

All datasets generated for this study are included in the article/**Supplementary Material**. Access to the code and the hydroponic experiments dataset can be obtained by contacting MK. Access to the the field trial dataset can be obtained by contacting AM.

## Author Contributions

MK has developed the model, analyzed the results, and prepared the manuscript. GT has provided insight on optimization modelling and sensitivity analysis. BR and SP have provided ideas on experiment design. AM has provided the field dataset. GT, SP, PC, DG, AM and BR have provided commentaries on the drafted paper.

## Conflict of Interest

The authors declare that the research was conducted in the absence of any commercial or financial relationships that could be construed as a potential conflict of interest.

## References

[B1] Ågren G. I. Ingestad T. (1987). Root: shoot ratio as a balance between nitrogen productivity and photosynthesis. Plant Cell Environ. 10 (7), 579–586. 10.1111/j.1365-3040.1987.tb01838.x

[B2] Ågren G. I. Wikström F. (1993). Modelling carbon allocation–a review. NZJ For. Sci. 23, 343–353.

[B3] Ågren G. I. (2008). Stoichiometry and nutrition of plant growth in natural communities. Annu. Rev. Ecol. Evol. Syst. 39 (1), 153–170. 10.1146/annurev.ecolsys.39.110707.173515

[B4] Agren G. I. (2003). Root: shoot ratios, optimization and nitrogen productivity. Ann. Bot. 92 (6), 795–800. 10.1093/aob/mcg203 14565938 PMC4243620

[B5] Amos B. Walters D. T. (2006). Maize root biomass and net Rhizodeposited carbon. Soil Sci. Soc. America J. 70 (5), 1489–1503. 10.2136/sssaj2005.0216

[B6] Andersen M. S. Dahl J. Vandenberghe L. (2018). CVXOPT: A Python Package for Convex Optimization (Version 1.2). https://cvxopt.org/.

[B7] Andre M. Massimino D. Daguenet A. (1978). Daily patterns under the life cycle of a maize crop: I. photosynthesis, transpiration, respiration. Physiol. Plant. 43 (4), 397–403. 10.1111/j.1399-3054.1978.tb01602.x

[B8] Anten N. P. R. During H. J. (2011). Is analysing the nitrogen use at the plant canopy level a matter of choosing the right optimization criterion? Oecologia 167 (2), 293–303. 10.1007/s00442-011-2011-3 21567246 PMC3172407

[B9] Asher C. J. Loneragan J. F. (1967). Response of plants to phosphate concentration in solution culture: I. growth and phosphorus content. Soil Sci. 103 (4), 225–233. 10.1097/00010694-196704000-00001

[B10] Atkin O. (2003). Thermal acclimation and the dynamic response of plant respiration to temperature. Trends Plant Sci. 8 (7), 343–351. 10.1016/S1360-1385(03)00136-5 12878019

[B11] Ball J. T. Woodrow I. E. Berry J. A. (1987). “A Model Predicting Stomatal Conductance and Its Contribution to the Control of Photosynthesis under Different Environmental Conditions,” in Progress in Photosynthesis Research (Dordrecht, Netherlands: Springer), 221–224.

[B12] Barber S. A. (1995). Soil Nutrient Bioavailability: A Mechanistic Approach. (New York, USA: John Wiley & Sons).

[B13] Berry J. Bjorkman O. (1980). Photosynthetic response and adaptation to temperature in higher plants. Annu. Rev. Plant Physiol. 31 (1), 491–543. 10.1146/annurev.pp.31.060180.002423

[B14] Bloom A. J. Chapin F S. III Mooney H. A. (1985). Resource limitation in plants-an economic analogy. Annu. Rev. Ecol. Syst. 16 (1), 363–392. 10.1146/annurev.es.16.110185.002051

[B15] Cakmak I. Hengeler C. Marschner H. (1994). Partitioning of shoot and root dry matter and Carbohydrates in bean plants suffering from Phosphorus, Potassium and Magnesium deficiency. J. Exp. Bot. 45 (9), 1245–1250. 10.1093/jxb/45.9.1245

[B16] Chapin F S. III Matson P. A. Vitousek P. (2011). Principles of Terrestrial Ecosystem Ecology (New York, USA: Springer Science & Business Media).

[B17] Collatz G. J. Ribas-Carbo M. Berry J. A. (1992). Coupled photosynthesis-stomatal conductance model for leaves of C4 plants. Funct. Plant Biol. 19 (5), 519–538. 10.1071/PP9920519

[B18] Dantzig G. B. Thapa M. N. (1997). Linear Programming. en. Springer Series in Operations Research (New York: Springer).

[B19] Daroub S. H. Gerakis A. Ritchie J. T. Friesen D. K. Ryan J (2003). Development of a soil-plant phosphorus simulation model for calcareous and weathered tropical soils. Agric. Syst. 76 (3), 1157–1181. 10.1016/S0308-521X(02)00082-3

[B20] De Kauwe M. G. Medlyn B. E. Zaehle S. Walker A. P. Dietze M. C. Wang Y.-P. (2014). Where does the carbon go? a model–data intercomparison of vegetation carbon allocation and turnover processes at two temperate forest free-air CO2 enrichment sites. New Phytol. 203 (3), 883–899. 10.1111/nph.12847 24844873 PMC4260117

[B21] De Vries F. W. T. P. Witlage J. M. Kremer D. (1979). Rates of respiration and of increase in structural dry matter in young wheat, ryegrass and maize plants in relation to temperature, to water stress and to their sugar content. Ann. Bot. 44 (5), 595–609. 10.1093/oxfordjournals.aob.a085772

[B22] De Vries F. W. T. P. (1972). “Respiration and growth,” in Crop Processes in Controlled Environments. (Cambridge, UK: Academic Press), 327–347.

[B23] Delve R. J. Probert M. E. Cobo J. G. Ricaurte J. Rivera F. Barrios E. (2009). Simulating phosphorus responses in annual crops using APSIM: model evaluation on contrasting soil types. Nutr. Cycling Agroecosyst. 84 (3), 293–306. 10.1007/s10705-008-9243-6

[B24] Dewar R. C. Franklin O. Mäkelä A. McMurtrie R. E. Valentine H. T (2009). Optimal function explains forest responses to global change. BioScience 59 (2), 127–139. 10.1525/bio.2009.59.2.6

[B25] Dzotsi K. A. Jones J. W. Adiku S. G. K. Naab J. B. Singh U. Porter C. H. (2010). Modeling soil and plant phosphorus within DSSAT. Ecol. Modell. 221 (23), 2839–2849. 10.1016/j.ecolmodel.2010.08.023

[B26] Elser J. J. Bracken M. E. S. Cleland E. E. Gruner D. S. Harpole W. S. Hillebrand H. (2007). Global analysis of nitrogen and phosphorus limitation of primary producers in freshwater, marine and terrestrial ecosystems. Ecol. Lett. 10 (12), 1135–1142. 10.1111/j.1461-0248.2007.01113.x 17922835

[B27] Elser J. J. Fagan W. F. Kerkhoff A. J. Swenson N. G. Enquist B. J (2010). Biological stoichiometry of plant production: metabolism, scaling and ecological response to global change: Tansley review. New Phytol. 186 (3), 593–608. 10.1111/j.1469-8137.2010.03214.x 20298486

[B28] Fageria N. K. Baligar V. C. (1989). Response of Legumes and cereals to Phosphorus in solution culture. J. Plant Nutr. 12 (9), 1005–1019. 10.1080/01904168909364019

[B29] Farquhar G. D. von Caemmerer S. Berry J. A. (2001). Models of photosynthesis. Plant Physiol. 125 (1), 42–45. 10.1104/pp.125.1.42 11154292 PMC1539321

[B30] Franklin O. McMurtrie R. E. Iversen C. M. Crous K. Y. Finzi A. C. Tissue D. T. (2009). Forest fine-root production and nitrogen use under elevated CO2: contrasting responses in evergreen and deciduous trees explained by a common principle. Global Change Biol. 15 (1), 132–144. 10.1111/j.1365-2486.2008.01710.x

[B31] Franklin O. Johansson J. Dewar R. C. Dieckmann U. McMurtrie R. E. Brannstrom A. (2012). Modeling carbon allocation in trees: a search for principles. Tree Physiol. 32 (6), 648–666. 10.1093/treephys/tpr138 22278378

[B32] Fredeen A. L. Rao I. M. Terry N. (1989). Influence of Phosphorus nutrition on growth and carbon partitioning in *Glycine Max* . Plant Physiol. 89 (1), 225–230. 10.1104/pp.89.1.225 16666518 PMC1055823

[B33] Fredeen A. L. Raab T. K. Rao I. M. Terry N (1990). Effects of phosphorus nutrition on photosynthesis in Glycine Max (L.) Merr. Planta 181 (3), 399–405. 10.1007/BF00195894 24196818

[B34] Goll D. S. Brovkin V. Parida B. R. Reick C. H. Kattge J. Reich P. B. (2012). Nutrient limitation reduces land carbon uptake in simulations with a model of combined Carbon, Nitrogen and Phosphorus cycling. Biogeosciences 9 (9), 3547–3569. 10.5194/bg-9-3547-2012

[B35] Goll D. S. Vuichard N. Maignan F. Jornet-Puig A. Sardans J. Violette A. (2017). A representation of the Phosphorus cycle for ORCHIDEE (Revision 4520). Geosci. Model Dev. 10 (10), 3745–3770. 10.5194/gmd-10-3745-2017

[B36] Gutschick V. P. (1988). Optimization of specific leaf mass, internal CO2 concentration and Chlorophyll content in crop canopies. Plant Physiol. Biochem. 26 (4), 525–537.

[B37] Heinen M. Mollier A. De Willigen P. (2003). Growth of a root system described as diffusion. II. Numerical model and application. Plant Soil 252 (2), 251–265. 10.1023/a:1024749022761

[B38] Herman J. D. Usher W. (2017). SALib: an open-source python library for sensitivity analysis. J. Open Source Softw. 2 (9), 97. 10.21105/joss.00097

[B39] Hinsinger P. Brauman A. Devau N. Gérard F. Jourdan C. Laclau J.-P. (2011). Acquisition of Phosphorus and other poorly mobile nutrients by roots. Where do plant nutrition models fail? Plant Soil 348 (1-2), 29–61. 10.1007/s11104-011-0903-y

[B40] Hsiao T. C. (1973). Plant responses to water stress. Annu. Rev. Plant Physiol. 24 (1), 519–570. 10.1146/annurev.pp.24.060173.002511

[B41] Iwasa Y. Roughgarden J. (1984). Shoot/root balance of plants: optimal growth of a system with many vegetative organs. Theor. Popul. Biol. 25 (1), 78–105. 10.1016/0040-5809(84)90007-8

[B42] Jacob J. Lawlor D. W. (1991). Stomatal and Mesophyll limitations of photosynthesis in Phosphate deficient sunflower, maize and wheat plants. J. Exp. Bot. 42 (8), 1003–1011. 10.1093/jxb/42.8.1003

[B43] Jones C. A. Cole C. V. Sharpley A. N. Williams C. A (1984). A simplified soil and plant phosphorus model: i. documentation 1. Soil Sci. Soc. America J. 48 (4), 800–805. 10.2136/sssaj1984.03615995004800040020x

[B44] Jones E. Oliphant T. Peterson P. (2001). SciPy: open source scientific tools for Python.

[B45] Kim S.-H. Sicher R. C. Bae H. Gitz D. C. Baker J. T. Timlin D. J. (2006). Canopy photosynthesis, evapotranspiration, leaf Nitrogen, and transcription profiles of Maize in response to CO2 enrichment. Global Change Biol. 12 (3), 588–600. 10.1111/j.1365-2486.2006.01110.x

[B46] Krinner G. Viovy N. de Noblet-Ducoudré N. Ogée J. Polcher J. Friedlingstein P. (2005). A dynamic global vegetation model for studies of the coupled atmosphere-biosphere system. Global Biogeochem. Cycles 19 (1), GB1015. 10.1029/2003GB002199

[B47] Lindquist J. L. Arkebauer T. J. Walters D. T. Cassman K. G. Dobermann A (2005). Maize radiation use efficiency under optimal growth conditions. Agron. J. 97 (1), 72–78. 10.2134/agronj2005.0072

[B48] Loneragan J. F. Grove T. S. Robson A. D. Snowball K (1979). Phosphorus toxicity as a factor in Zinc-Phosphorus interactions in plants. Soil Sci. Soc. Am. J. 43 (5), 966–972. 10.2136/sssaj1979.03615995004300050031x

[B49] Mäkelä A. Valentine H. T. Helmisaari H.-S. (2008). Optimal co-allocation of carbon and nitrogen in a forest stand at steady state. New Phytol. 180 (1), 114–123. 10.1111/j.1469-8137.2008.02558.x 18637066

[B50] Marschner H. Kirkby E. A. Cakmak I. (1996). Effect of mineral nutritional status on shoot-root partitioning of Photoassimilates and cycling of mineral nutrients. J. Exp. Bot. 47 (Special_Issue), 1255–1263. 10.1093/jxb/47.Special_Issue.1255 21245257

[B51] McMurtrie R. E. Norby R. J. Medlyn B. E. Dewar R. C. Pepper D. A. Reich P. B. (2008). Why is plant-growth response to elevated CO2 amplified when water is limiting, but reduced when Nitrogen is limiting? A growth-optimisation hypothesis. Funct. Plant Biol. 35 (6), 521–534. 10.1071/FP08128 32688808

[B52] Meir P. Grace J. Miranda A. C. (2001). Leaf respiration in two tropical rainforests: constraints on physiology by Phosphorus, Nitrogen and temperature. Funct. Ecol. 15 (3), 378–387. 10.1046/j.1365-2435.2001.00534.x

[B53] Niu Y. F. Chai R. S. Jin G. L. Wang H. Tang C. X. Zhang Y. S (2013). Responses of root architecture development to low phosphorus availability: a review. Ann. Bot. 112 (2), 391–408. 10.1093/aob/mcs285 23267006 PMC3698383

[B54] Nossent J. Elsen P. Bauwens W. (2011). Sobol' sensitivity analysis of a complex environmental model. Environ. Modell. Softw. 26 (12), 1515–1525. 10.1016/j.envsoft.2011.08.010

[B55] Parton W. J. Stewart J. W. B. Cole C. V. (1988). Dynamics of C, N, P and S in grassland soils: a model. Biogeochemistry 5 (1), 109–131. 10.1007/BF02180320

[B56] Peñuelas J. Poulter B. Sardans J. Ciais P. van der Velde M. Bopp L. (2013). Human-induced Nitrogen–Phosphorus imbalances alter natural and managed ecosystems across the globe”. en. Nat. Commun. 4 (1), 2934. 10.1038/ncomms3934 24343268

[B57] Plénet D. Mollier A. Pellerin S. (2000). Growth analysis of Maize field crops under phosphorus deficiency. II. radiation-use efficiency, biomass accumulation and yield components. Plant Soil 224 (2), 259–272. 10.1023/A:1004835621371

[B58] Plaxton W. Lambers H. (2015). Annual Plant Reviews, Phosphorus Metabolism in Plants Vol. 48 (New York, USA: John Wiley & Sons).

[B59] Poorter H. Niklas K. J. Reich P. B. Oleksyn J. Poot P. Mommer L (2012). Biomass allocation to leaves, stems and roots: meta-analyses of interspecific variation and environmental control: Tansley review. New Phytol. 193 (1), 30–50. 10.1111/j.1469-8137.2011.03952.x 22085245

[B60] Ringeval B. Augusto L. Monod H. van Apeldoorn D. Bouwman L. Yang X. (2017). Phosphorus in agricultural soils: drivers of its distribution at the global scale. Global Change Biol. 23 (8), 3418–3432. 10.1111/gcb.13618 28067005

[B61] Rosenzweig C. Elliott J. Deryng D. Ruane A. C. Müller C. Arneth A. (2014). Assessing agricultural risks of climate change in the 21st Century in a global gridded crop model intercomparison. Proc. Natl. Acad. Sci. 111 (9), 3268–3273. 10.1073/pnas.1222463110 24344314 PMC3948251

[B62] Rowland L. Zaragoza-Castells J. Bloomfield K. J. Turnbull M. H. Bonal D. Burban B. (2017). Scaling leaf respiration with nitrogen and phosphorus in tropical forests across two continents. New Phytol. 214 (3), 1064–1077. 10.1111/nph.13992 27159833 PMC5412872

[B63] Ruimy A. Dedieu G. Saugier B. (1996). TURC: a diagnostic model of continental gross primary productivity and net primary productivity. Global Biogeochem. Cycles 10 (2), 269–285. 10.1029/96GB00349

[B64] Ryan M. G. Hubbard R. M. Pongracic S. Raison R. J. McMurtrie R. E (1996). Foliage, fine-root, woody-tissue and stand respiration in pinus radiata in relation to nitrogen status. Tree Physiol. 16 (3), 333–343. 10.1093/treephys/16.3.333 14871734

[B65] Ryan P. R. Delhaize E. Watt M. Richardson A. E (2016). Plant roots: understanding structure and function in an ocean of complexity. Ann. Bot. 118 (4), 555–559. 10.1093/aob/mcw192

[B66] Rychter A. M. Randall D. D. (1994). The effect of phosphate deficiency on carbohydrate metabolism in bean roots. Physiol. Plant. 91 (3), 383–388. 10.1111/j.1399-3054.1994.tb02964.x

[B67] Schieving F. Poorter H. (1999). Carbon gain in a multispecies canopy: the role of specific leaf area and photosynthetic nitrogen-use efficiency in the tragedy of the commons. New Phytol. 143 (1), 201–211. 10.1046/j.1469-8137.1999.00431.x

[B68] Shinozaki K. Yoda K. Hozumi K. Kira K (1964). A quantitative analysis of plant form-the pipe model theory: I. basic analyses. Jpn. J. Ecol. 14 (3), 97–105. 10.18960/seitai.14.3_97

[B69] Sobol I. M. (1993). Sensitivity estimates for nonlinear mathematical models. Math. Modell. Comput. Exp. 1 (4), 407–414.

[B70] Sterck F. J. Poorter L. Schieving F. (2006). Leaf traits determine the growth-survival trade-off across rain forest tree species. Am. Nat. 167 (5), 758–765. 10.1086/503056 16671019

[B71] Thornley J. H. M. (1976). Mathematical Models in Plant Physiology (London, UK: Academic Press (Inc.) London, Ltd.).

[B72] Thornley J. H. M. (1995). Shoot: root allocation with respect to C, N and P: an investigation and comparison of resistance and teleonomic models. Ann. Bot. 75 (4), 391–405. 10.1006/anbo.1995.1037

[B73] Vitousek P. M. Porder S. Houlton B. Z. Chadwick O. A (2010). Terrestrial phosphorus limitation: mechanisms, implications, and nitrogen–phosphorus interactions. Ecol. Appl. 20 (1), 5–15. 10.1890/08-0127.1 20349827

[B74] Wang Y.-P. Houlton B. Z. Field C. B. (2007). A model of biogeochemical cycles of carbon, nitrogen, and phosphorus including symbiotic nitrogen fixation and phosphatase production: a biogeochemical model of C, N, and P. Global Biogeochem. Cycles 21 (1), GB1018. 10.1029/2006GB002797

[B75] Wang Y. P. Law R. M. Pak B. (2010). A global model of carbon, nitrogen and phosphorus cycles for the terrestrial biosphere. Biogeosciences 7 (7), 2261–2282. 10.5194/bg-7-2261-2010

[B76] Yang X. Thornton P. E. Ricciuto D. M. Post W. M (2014). The role of phosphorus dynamics in tropical forests – a modeling study using CLM-CNP. Biogeosciences 11 (6), 1667–1681. 10.5194/bg-11-1667-2014

[B77] Yang X. Ricciuto D. M. Thornton P. E. Shi X. Xu M. Hoffman F. (2019). The effects of phosphorus cycle dynamics on carbon sources and sinks in the amazon region: a modeling study using ELM V1. J. Geophys. Res.: Biogeosci. 124, 3686–3698. 10.1029/2019jg005082

[B78] Zaehle S. Dalmonech D. (2011). Carbon–nitrogen interactions on land at global scales: current understanding in modelling climate biosphere feedbacks. Curr. Opin. In Environ. Sustain. 3 (5), 311– 320. 10.1016/j.cosust.2011.08.008

