# Supplementary material for: Carbon and Phosphorus Allocation in Annual Plants: An Optimal Functioning Approach
Source: Front Plant Sci. 2020 Feb 27;11:149. doi: 10.3389/fpls.2020.00149 (PMC7056904; doi:10.3389/fpls.2020.00149)
Supplement: Supplementary file 1 [file DataSheet_1.pdf]

Supplementary information to :

Carbon and phosphorus allocation in annual plants: an optimal functioning approach

Marko Kvakić[1,2,*], George Tzagkarakis[3], Sylvain Pellerin[1], Philippe Ciais[2], Daniel Goll[2], Alain Mollier[1], and Bruno Ringeval[1]

[1]*ISPA, Bordeaux Sciences Agro, INRA, 33140 Villenave d'Ornon, France*

[2]*LSCE/IPSL, CEA-CNRS-UVSQ, Universite Paris-Saclay, F-91191, Gif-sur-Yvette, France*

[3]*ICS - Foundation for Research & Technology - Hellas (FORTH), Crete, Greece*

[*]*For correspondece. E-mail: markokvakic@gmail.com*

December 15, 2019

1 # 1 Supplementary Tables

Supplementary Table 1: Calibrated values for the maize field trial. Parameter k_{CR} is given for each of the 12 P experiments (3 P levels x 4 blocks) and all others kept the same. Parameter description can be found in Tables 1 and 2.

Parameter	Initial guess	Calibrated values ± std. error
k_{CL}	1.5	1.7137 ± 0.1106
$k_{CR,1}$	1.0	0.9101 ± 0.1017
$k_{CR,2}$	1.0	0.8302 ± 0.0922
$k_{CR,3}$	1.0	0.8545 ± 0.0925
$k_{CR,4}$	1.0	0.8376 ± 0.0969
$k_{CR,5}$	1.0	1.4577 ± 0.1673
$k_{CR,6}$	1.0	1.4526 ± 0.1529
$k_{CR,7}$	1.0	1.1963 ± 0.1111
$k_{CR,8}$	1.0	1.4287 ± 0.1573
$k_{CR,9}$	1.0	1.7878 ± 0.2064
$k_{CR,10}$	1.0	1.8376 ± 0.1784
$k_{CR,11}$	1.0	1.7906 ± 0.1966
$k_{CR,12}$	1.0	1.8377 ± 0.1859
$C_{L,max}$	1.5	0.9042 ± 0.0862
$\rho_{L,max}$	1.0	1.0927 ± 0.0904
λ_{CR}	0.30	0.1362 ± 0.0291
λ_{CL}	0.10	0.1608 ± 0.0141
λ_{CS}	0.03	0.0117 ± 0.0020
λ_{CG}	0.01	0.0153 ± 0.0048
f_{CS}	0.5	0.4036 ± 0.0495
f_{CG}	1.0	2.3523 ± 0.2097
f_{PS}	0.8	1.5956 ± 0.0660
f_{PR}	0.1	0.0399 ± 0.0850
k_{mPL}	0.05	0.0584 ± 0.0081
k_{mPR}	0.05	0.2541 ± 0.1193
k_{mPS}	0.05	0.0686 ± 0.0096
$C_{L,0}$	0.1	0.0064 ± 0.0250
$P_{L,0}$	0.1	0.0298 ± 0.0180

Supplementary Table 2: Calibrated parameter values for the hydroponic studies. Parameter k_{CR} is given for each of the five P experiments and all others kept the same for each species (Asher and Loneragan, 1967; Fageria and Baligar, 1989). Parameter description can be found in Tables 1 and 2.

Variable	k_{CL}	$C_{L,max}$	$\rho_{L,max}$	λ_{CR}	λ_{CL}	$C_{L,0}$
Initial guess	1.5	1.0	1.5	0.3	0.1	0.1
Barrel medic	1.2217 ± 1.2479	1.4816 ± 0.9336	0.8967 ± 0.2541	0.3261 ± 0.4065	0.1195 ± 0.1727	0.0441 ± 0.2109
Brome grass	1.8221 ± 0.5271	0.4289 ± 0.0644	1.2571 ± 0.2453	0.4145 ± 0.1946	0.1187 ± 0.0651	0.1211 ± 0.4818
Capeweed	1.2570 ± 0.5783	1.6330 ± 1.1550	0.9148 ± 0.4691	0.2489 ± 1.2535	0.1224 ± 0.4597	0.0524 ± 0.8952
Clover	1.7286 ± 0.3377	0.4744 ± 0.1035	1.1299 ± 0.2715	0.4721 ± 0.1357	0.0847 ± 0.0482	0.1395 ± 0.1960
Erodium	2.0614 ± 2.4349	0.7016 ± 1.0327	1.1258 ± 1.3077	0.6225 ± 1.1166	0.0720 ± 0.3201	0.0956 ± 0.5452
Flatweed	2.0698 ± 0.6796	0.4879 ± 0.1347	2.0429 ± 0.4540	0.4136 ± 0.2458	0.1366 ± 0.1897	0.1208 ± 0.2399
Silver grass	1.7724 ± 0.7744	0.4667 ± 0.2487	1.4596 ± 1.0746	0.4734 ± 0.7083	0.1048 ± 0.2218	0.1309 ± 0.4042
Alfalfa	1.7676 ± 1.0372	0.4844 ± 0.1900	1.7205 ± 0.4770	0.4672 ± 0.5006	0.1132 ± 0.2824	0.1419 ± 0.4264
Bean	1.4107 ± 0.4049	0.3595 ± 0.0642	1.3110 ± 0.2805	0.5136 ± 0.3943	0.1099 ± 0.1339	0.1300 ± 0.3123
Red clover	1.5970 ± 1.4740	0.3258 ± 0.2304	1.9161 ± 0.7067	0.3124 ± 0.3857	0.1165 ± 0.1431	0.0873 ± 0.5166
Rice	1.4473 ± 0.9212	0.1676 ± 0.0903	3.7253 ± 1.1436	0.3411 ± 1.1679	0.1157 ± 0.6166	0.0858 ± 0.8162
Wheat	1.7524 ± 0.7460	0.6345 ± 0.2700	1.2003 ± 0.4162	0.3365 ± 0.3818	0.1091 ± 0.2223	0.0447 ± 0.5497

Variable	$P_{L,0}$	$k_{CR,1}$	$k_{CR,2}$	$k_{CR,3}$	$k_{CR,4}$	$k_{CR,5}$
Initial guess	0.1	1.0	1.0	1.0	1.0	1.0
Barrel medic	0.0214 ± 0.5136	0.1588 ± 3.3886	0.6731 ± 2.0856	0.8325 ± 1.6676	1.1213 ± 1.0650	1.2884 ± 0.7138
Brome grass	0.0723 ± 0.2866	0.0360 ± 0.1446	0.2408 ± 0.2552	1.1367 ± 0.3647	1.3340 ± 0.4073	1.8140 ± 0.3765
Capeweed	0.0609 ± 0.3320	0.2978 ± 0.4454	0.4815 ± 0.5419	0.5965 ± 0.6870	0.8538 ± 0.8076	1.1816 ± 0.9310
Clover	0.1203 ± 0.2558	0.0348 ± 0.0862	0.2690 ± 0.1442	1.1347 ± 0.3132	2.2824 ± 0.6717	2.0929 ± 0.5477
Erodium	0.0415 ± 1.6314	0.0215 ± 1.1265	0.3436 ± 2.0639	0.7719 ± 2.3206	1.6700 ± 2.8556	1.8104 ± 3.0153
Flatweed	0.0961 ± 0.4203	0.0782 ± 0.1879	0.3508 ± 0.1543	0.9953 ± 0.1750	1.6565 ± 0.4316	1.4294 ± 0.2642
Silver grass	0.1066 ± 1.3494	0.0809 ± 0.5959	0.3281 ± 0.6141	0.7678 ± 0.4249	1.8235 ± 0.6355	1.7826 ± 0.8745
Alfalfa	0.0713 ± 0.8038	0.0259 ± 0.4894	0.2313 ± 0.4821	0.9014 ± 0.3704	1.5848 ± 0.3303	2.1503 ± 0.6900
Bean	0.0464 ± 0.1495	0.0630 ± 0.1667	0.5332 ± 0.2332	0.8130 ± 0.2835	1.2538 ± 0.3362	1.4391 ± 0.3720
Red clover	0.0719 ± 0.7136	0.1330 ± 0.5427	0.4411 ± 0.5719	0.6859 ± 0.4559	0.8600 ± 0.5091	1.0676 ± 0.4154
Rice	0.0468 ± 0.4628	0.0548 ± 0.8097	0.4648 ± 0.5638	1.4896 ± 0.9318	1.2552 ± 0.7613	1.2643 ± 0.8733
Wheat	0.0440 ± 0.3336	0.1627 ± 0.4041	0.2031 ± 0.4443	0.5298 ± 0.6615	0.8987 ± 1.0080	1.0202 ± 0.9067

Supplementary Table 3: Calibrated parameter values for the Michaelis-Menten kinetic (Eq. 15). a_{C_P} unit is kgP t^{-1}DW root ha^{-1} day^{-1} in the field maize trial, and mgP g^{-1}DW root pot^{-1} day^{-1} in the hydroponic studies. b_{C_P} unit is mgP L^{-1} in the field maize trial, and μP L^{-1} in the hydroponic studies.

Scale	Species	a_{C_P}	b_{C_P}
Field	Maize	2.13 ± 0.12	0.78 ± 0.13
Hydroponic	Barrel medic	1.72 ± 0.06	0.74 ± 0.13
	Brome grass	2.39 ± 0.09	0.88 ± 0.18
	Capeweed	1.99 ± 0.04	1.31 ± 0.11
	Clover	1.63 ± 0.05	0.51 ± 0.12
	Erodium	2.03 ± 0.07	1.21 ± 0.20
	Flatweed	2.22 ± 0.04	1.68 ± 0.11
	Silver grass	1.49 ± 0.09	0.23 ± 0.07
Hydroponic	Alfalfa	1.46 ± 0.04	64.78 ± 5.77
	Bean	1.24 ± 0.17	67.24 ± 30.39
	Red clover	1.95 ± 0.06	126.51 ± 10.55
	Rice	1.25 ± 0.05	80.83 ± 9.96
	Wheat	1.72 ± 0.27	227.72 ± 73.89

2 Supplementary Figures

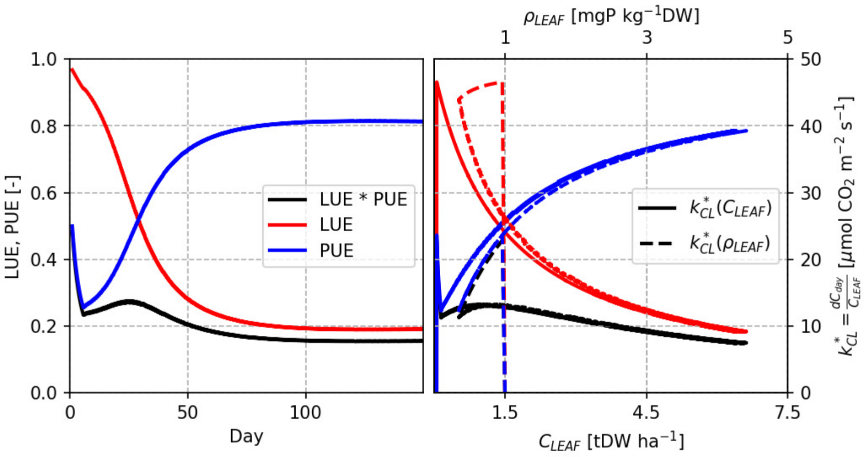

Supplementary Figure 1: Time evolution of leaf LUE and PUE (left) and the effective assimilation rate (k^*_{CL}, right) as function of leaf biomass and concentration. Root uptake rate k_{CR} is set to 1.0 kgP tDW^{-1} root ha^{-1} day^{-1}.

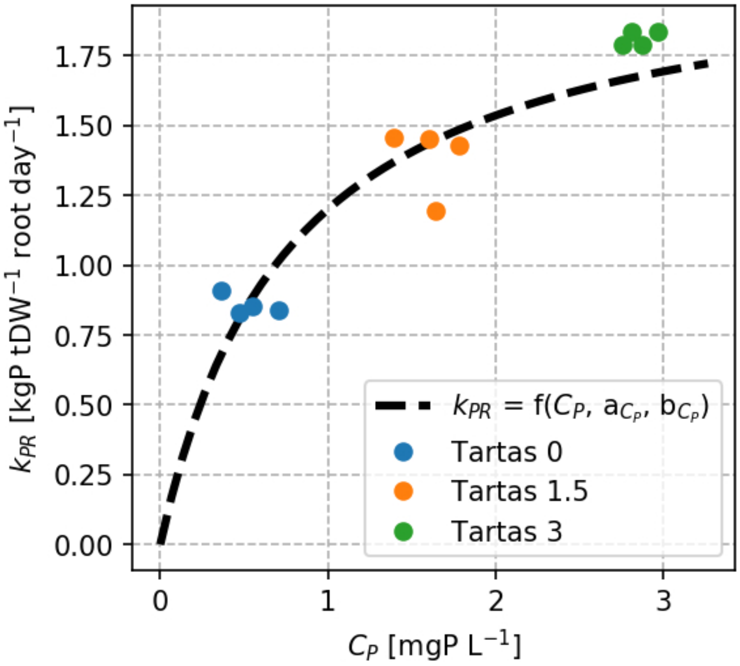

Supplementary Figure 2: Predicted field trial root P uptake (k_{CR}) vs. measured soil solution P concentration. Different markers and colors depict different levels of P addition. Dark dashed line depicts a Michaelis-Menten kinetic (similar to Eq. 15) which best describes the relationship between the two.

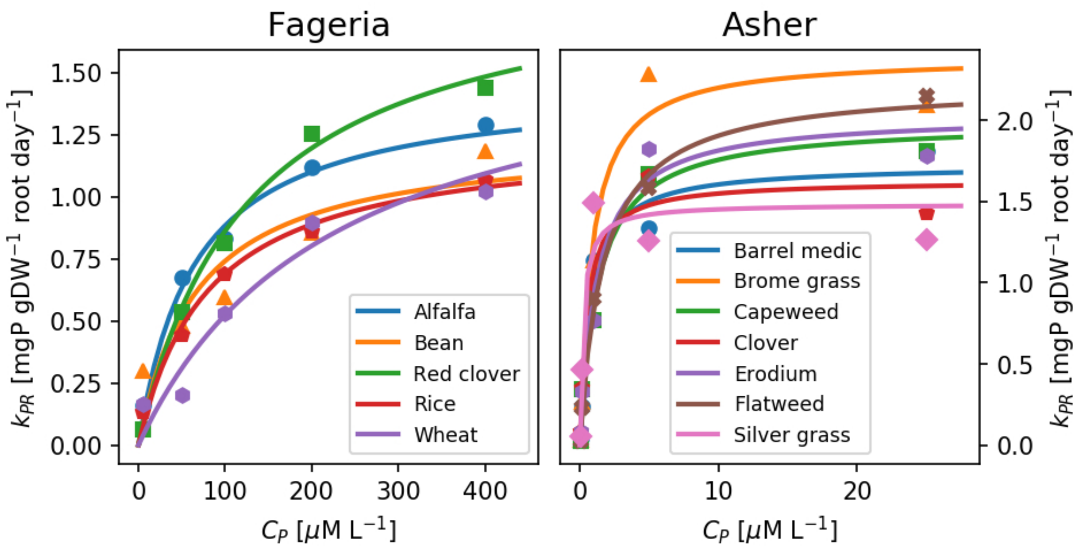

Supplementary Figure 3: Predicted hydroponic studies root P uptake (k_{CR}) vs. measured solution P concentration. Different markers and colors depict different levels of P addition. Dark dashed line depicts a Michaelis-Menten kinetic (similar to Eq. 15) which best describes the relationship between the two.
